# Effect of Medication Optimization vs Cognitive Behavioral Therapy Among US Veterans With Chronic Low Back Pain Receiving Long-term Opioid Therapy: A Randomized Clinical Trial

**DOI:** 10.1001/jamanetworkopen.2022.42533

**Published:** 2022-11-17

**Authors:** Michael A. Bushey, James E. Slaven, Samantha D. Outcalt, Kurt Kroenke, Carol Kempf, Amanda Froman, Christy Sargent, Brad Baecher, Alan J. Zillich, Teresa M. Damush, Chandan Saha, Dustin D. French, Matthew J. Bair

**Affiliations:** 1 Department of Psychiatry, Indiana University School of Medicine, Indianapolis; 2 Department of Biostatistics and Heath Data Science, Indiana University School of Medicine, Indianapolis; 3 Veterans Affairs (VA) Health Services Research & Development Center for Health Information and Communication, Roudebush VA Medical Center, Indianapolis, Indiana; 4 Department of Medicine, Indiana University School of Medicine, Indianapolis; 5 Regenstrief Institute, Inc, Indianapolis, Indiana; 6 Department of Pharmacy Practice, Purdue University College of Pharmacy, West Lafayette, Indiana; 7 Department of Ophthalmology, Medical Social Sciences, Northwestern University Feinberg School of Medicine, Chicago, Illinois; 8 Department of Medical Social Sciences, Northwestern University Feinberg School of Medicine, Chicago, Illinois; 9 Center for Health Services and Outcomes Research, Chicago, Illinois; 10 Center of Innovation for Complex Chronic Healthcare, Edward Hines Jr VA Hospital, Hines, Illinois

## Abstract

**Importance:**

Medication management and cognitive behavioral therapy (CBT) are commonly used treatments for chronic low back pain (CLBP). However, little evidence is available comparing the effectiveness of these approaches.

**Objective:**

To compare collaborative care medication optimization vs CBT on pain intensity, interference, and other pain-related outcomes.

**Design, Setting, and Participants:**

The Care Management for the Effective Use of Opioids (CAMEO) trial was a 12-month, comparative effectiveness randomized clinical trial with blinded outcome assessment. Recruitment of veterans with CLBP prescribed long-term opioids occurred at 7 Veterans Affairs primary care clinics from September 1, 2011, to December 31, 2014, and follow-up was completed December 31, 2015. Analyses were based on intention to treat in all randomized participants and were performed from March 22, 2015, to November 1, 2021.

**Interventions:**

Patients were randomized to receive either collaborative care with nurse care manager–delivered medication optimization (MED group) (n = 131) or psychologist-delivered CBT (CBT group) (n = 130) for 6 months, with check-in visits at 9 months and final outcome assessment at 12 months.

**Main Outcomes and Measures:**

The primary outcome was change in Brief Pain Inventory (BPI) total score, a composite of the pain intensity and interference subscales at 6 (treatment completion) and 12 (follow-up completion) months. Scores on the BPI range from 0 to 10, with higher scores representing greater pain impact and a 30% improvement considered a clinically meaningful treatment response. Secondary outcomes included pain-related disability, pain catastrophizing, self-reported substance misuse, health-related quality of life, depression, and anxiety.

**Results:**

A total of 261 patients (241 [92.3%] men; mean [SD] age, 57.9 [9.5] years) were randomized and included in the analysis. Baseline mean (SD) BPI scores in the MED and CBT groups were 6.45 (1.79) and 6.49 (1.67), respectively. Improvements in BPI scores were significantly greater in the MED group at 12 months (between-group difference, −0.54 [95% CI, −1.18 to −0.31]; *P* = .04) but not at 6 months (between-group difference, −0.46 [95% CI, −0.94 to 0.11]; *P* = .07). Secondary outcomes did not differ significantly between treatment groups.

**Conclusions and Relevance:**

In this randomized clinical trial among US veterans with CLBP who were prescribed long-term opioid therapy, collaborative care medication optimization was modestly more effective than CBT in reducing pain impact during the 12-month study. However, this difference may not be clinically meaningful or generalize to nonveteran populations.

**Trial Registration:**

ClinicalTrials.gov Identifier: NCT01236521

## Key Points

**Question:**

What are the most effective pain treatments for patients prescribed opioids?

**Findings:**

In this randomized clinical trial of 261 veterans with chronic low back pain prescribed opioids, pain improvement on the Brief Pain Inventory was greater with medication optimization (decrease of 1.10 points) than cognitive behavioral therapy (decrease of 0.68 points) for 12 months, a significant but clinically modest difference.

**Meaning:**

Both pharmacological and behavioral approaches are reasonable options for treating chronic low back pain in patients prescribed opioids.

## Teaser

This randomized clinical trial compares a care manager–delivered, pain medication optimization intervention with psychologist-delivered cognitive behavioral therapy for 12 months among US veterans with chronic low back pain who are receiving long-term opioid therapy.

## Introduction

Low back pain is the most common presenting condition in ambulatory care,^1,2^ accounting for significant patient disability, suffering, and decreased quality of life.^3^ Back pain is reported in more than half of patients prescribed long-term opioid therapy.^3,4^ Opioid prescribing rates quadrupled from 1999 to 2010,^5,6^ paralleled by increases in overdose deaths involving opioids.^7,8^ Opioids are more effective than placebo for short-term pain relief,^9^ are equally effective but more poorly tolerated than nonopioid analgesics in a head-to-head trial,^10^ and have not demonstrated effectiveness for periods longer than 12 months.^9^ Mitigation measures to decrease opioid prescribing through encouragements or mandates have led to decreased prescribing, although overdose deaths have continued to rise.^11^ Evidence-based approaches for pain treatment adjustment in patients receiving long-term opioid therapy are needed.

Collaborative care models—in which a care manager specialist team complements primary care to manage 1 or more target conditions—have consistently outperformed usual care in improving pain outcomes.^12,13,14,15^ These models have included step-based pharmacological algorithms, typically paired with nonpharmacological treatments. Although pharmacological treatments are most commonly used in clinical settings, nonpharmacological approaches are emphasized as first-line therapy in practice guidelines.^16,17^ Cognitive behavioral therapy (CBT) is a skills-based treatment that teaches patients coping skills^18^ and has the strongest evidence of nonpharmacological treatments for chronic pain.^19,20^ To our knowledge, no comparative effectiveness trials testing pharmacological vs nonpharmacological approaches to treat chronic low back pain (CLBP) have been published.

The Care Management for the Effective Use of Opioids (CAMEO) trial is a comparative effectiveness randomized clinical trial comparing a care manager–delivered, pain medication optimization intervention (MED group) with psychologist-delivered CBT (CBT group) for 12 months in primary care patients with CLBP prescribed long-term opioid therapy. We hypothesized that the CAMEO trial would detect a clinically meaningful difference in pain impact between study treatments at 6 and 12 months.

## Methods

### Study Participants

Details of the CAMEO randomized clinical trial have been described previously^21^ and are provided in the trial protocol in Supplement 1. Military veterans aged 18 years or older with CLBP of moderate severity (≥5 on a 10-point scale) and duration of at least 6 months prescribed long-term opioid therapy (≥3 opioid prescriptions of any dosage ≥28 days during the prior 12 months) and access to a working telephone were enrolled. Patients were recruited from 1 of 5 primary care clinics in the Roudebush Veterans Administration Medical Center (VAMC) and 2 community-based outpatient clinics (CBOCs) in Indiana. We excluded patients with significant cardiovascular disease, lung disease requiring home oxygen therapy, cancer in active or imminent treatment, pending back surgery, schizophrenia, active psychosis, active suicidal ideation, moderately severe cognitive impairment, active substance use disorder, active involvement in another pain trial, and current or expectant pregnancy.

Potential participants were identified by searching electronic medical records for CLBP codes in the *International Classification of Diseases, Ninth Revision* (721.x; 722.x, or 724.x), a primary care visit in the past 2 years, moderate pain severity, and long-term opioid prescription. Primary care clinician consent was obtained before approaching potentially eligible patients. Letters signed by the primary care clinician were mailed to potential participants describing the study, followed by telephone contact to assess eligibility. Interested patients who met eligibility criteria were scheduled for an appointment to obtain written informed consent and complete a baseline interview. Race and ethnicity and sex were determined by survey instrument at the baseline assessment as required by the funding agency. The trial was approved by the Indiana University Institutional Review Board and the Roudebush VAMC Research Review Committee. This report was prepared in accordance with the Consolidated Standards of Reporting Trials (CONSORT) reporting guideline.

### Randomization and Allocation Concealment

After providing consent and completing the interview, participants were randomized to collaborative care with a nurse care manager (NCM)–delivered medication optimization (MED group) or psychologist-delivered CBT (CBT group). Randomization used block sizes of 2, 4, 6, and 8 to ensure allocation concealment and treatment groups of equal size. The Master’s-level study statistician (J.E.S.) used a random-numbers table to generate the allocation sequence, stratified by study site (ie, VAMC vs CBOC). We did not stratify by history of depression or substance use disorder because we expected randomization to balance these potential confounders. Treatment assignments were supplied in sealed opaque envelopes to the study coordinators (A.F. and C.S.), who enrolled participants and assigned interventions. All baseline and follow-up assessments were conducted by a research assistant (B.B.) blinded to treatment allocation.

### Outcome Measures

Assessments were conducted at baseline and at 3, 6, 9, and 12 months. Pain was assessed with the Brief Pain Inventory (BPI),^14,22^ a validated measure that rates past-week pain severity (4 items) and pain interference (7 items). Scores on the BPI range from 0 to 10, with higher scores representing worse pain and a 1-point change considered clinically important.^23,24^ The primary outcome was the mean between-group differences (change scores from baseline to follow-up assessment) at 6 months (treatment completion) and 12 months in BPI total score, a composite of the pain severity and interference scores. Secondary outcomes included between-group comparisons during the 12-month trial of response rates (with individual response defined as 30% or greater decrease in BPI total from baseline)^24^; the Roland-Morris Disability Questionnaire (RMDQ)^25^; Pain Catastrophizing Scale^26^; Alcohol Use Disorders Identification Test–Concise^27^; Current Opioid Misuse Measure (COMM)^28^; 36-item Short Form Health Survey (SF-36) General Health, Social Functioning, and Vitality scales^29^; Patient Health Questionnaire 9-item Depression scale (PHQ-9)^30^; and Generalized Anxiety Disorder 7-item scale (GAD-7).^31^

Daily analgesic dose was collected as an exploratory secondary outcome. Analgesic information was obtained by retrospective extraction of prescription information from the electronic health record for each participant at each time point. Morphine milligram equivalents (MMEs) were calculated using the Center for Disease Control and Prevention’s opioid dose conversion table.^32^ A list of the analgesics surveyed can be found in eTable 1 in Supplement 2.

### Interventions

#### NCM-Administered Medication Management and Analgesic Optimization

At the baseline visit, the NCM assessed past and current treatments for CLBP. The NCMs delivered algorithm-based analgesic treatment along with guideline-concordant opioid management (as described hereinafter).^33^ Participants were scheduled to receive at least 8 treatment sessions lasting approximately 30 minutes each during 6 months and 1 check-in session at 9 months. During these contacts, NCMs assessed pain severity, treatment response, analgesic adherence, adverse effects, and desire to change treatment. The NCMs reviewed cases and treatment plans with physician and pharmacist investigators during weekly meetings. Analgesic prescription adjustments were made by the study team with communication to primary care clinicians through the electronic health record as appropriate.

The stepped-care analgesic algorithm was based on algorithms used in previous trials^13,15^ and is further described in the trial protocol in Supplement 1. Medication adjustments were guided by treatment response, adverse effects, and patient preference. Therapy with ineffective or poorly tolerated medications (including opioids) was reduced or eliminated when possible, and the following alternatives were added or optimized in the following sequence unless contraindicated (eg, avoiding nonsteroidal anti-inflammatory drugs in patients with kidney disease): (1) simple analgesics (acetaminophen and nonsteroidal anti-inflammatory drugs), (2) serotonin and norepinephrine reuptake inhibitors (duloxetine hydrochloride and venlafaxine hydrochoride), (3) gabapentinoids (gabapentin and pregabalin), (4) tricyclic antidepressants (nortriptyline hydrochloride and amitriptyline hydrochloride), (5) cyclobenzaprine, (6) tramadol hydrochloride, and (7) full-agonist opioids. All patients were asked to sign an opioid treatment agreement. Of note, opioid tapering was not an explicit goal of medication adjustments.

#### Psychologist-Led CBT

Participants randomized to the CBT group were scheduled to receive 8 individual treatment sessions delivered by clinical psychologists during 6 months. The sessions were delivered by telephone or face to face depending on patient preferences and scheduled at baseline; weeks 2, 4, 6, and 8; and months 3, 4 and 6, with a check-in session at 9 months. These 45-minute sessions consisted of 3 parts: (1) check-in (including update on pain, progress, and concerns), (2) intervention (barrier identification, skill learning, and practice), and (3) wrap-up (reflection, practice assignments, and goal setting). Intervention fidelity was addressed by having psychologists complete a training workshop, use treatment scripts, document treatment delivery details, and participate in weekly sessions with a supervising psychologist.

The manualized CBT intervention was augmented by a psychologist-led, one-on-one skills training program designed to increase self-efficacy in managing CLBP. Participants were introduced to a menu of skills, including pain education, relaxation, activity pacing, cognitive restructuring, self-care, interpersonal skills (patient-clinician communication), and pain flare relapse prevention. The final session at 9 months reinforced previously learned skills. This program evolved from validated materials proven effective in treating low back and arthritis pain in clinical trials performed by members of our team and others.^14,15,34,35,36,37^

### Statistical Analysis

Data were analyzed from March 22, 2015, to November 1, 2021. Initial reporting of sample size estimates for this trial on ClinicalTrials.gov and the statistical analysis plan (included in Supplement 1) contained mathematical errors. The correct sample size to have 80% power to detect a 0.3-SD between-group difference, assuming 12% attrition, would be 199 participants per group. After maximally extending our enrollment period, we enrolled a total of 261 participants, providing 62% power to detect a 0.3-SD difference given our 11% attrition rate. Between-group differences in outcomes during the 12 months of the trial were compared using multivariable mixed-effects models for repeated measures. Analyses were based on intention to treat in all randomized participants. Missing data were determined to be consistent with a missing at random hypothesis. The primary outcome models accounted for within-participant correlation for repeated measures using compound symmetric matrices (determined after analyzing several different appropriate structures) and adjusted for baseline outcome measure score as well as baseline depression, sex, and time. The models for secondary outcomes adjusted for baseline outcome measure score only. All analytic assumptions were verified, and analyses were performed using SAS, version 9.4 (SAS Institute Inc). Two-sided *P* < .05 was considered statistically significant. Quantitative data are reported as mean (SD) unless otherwise specified.

## Results

### Study Participants

**Figure 1.**
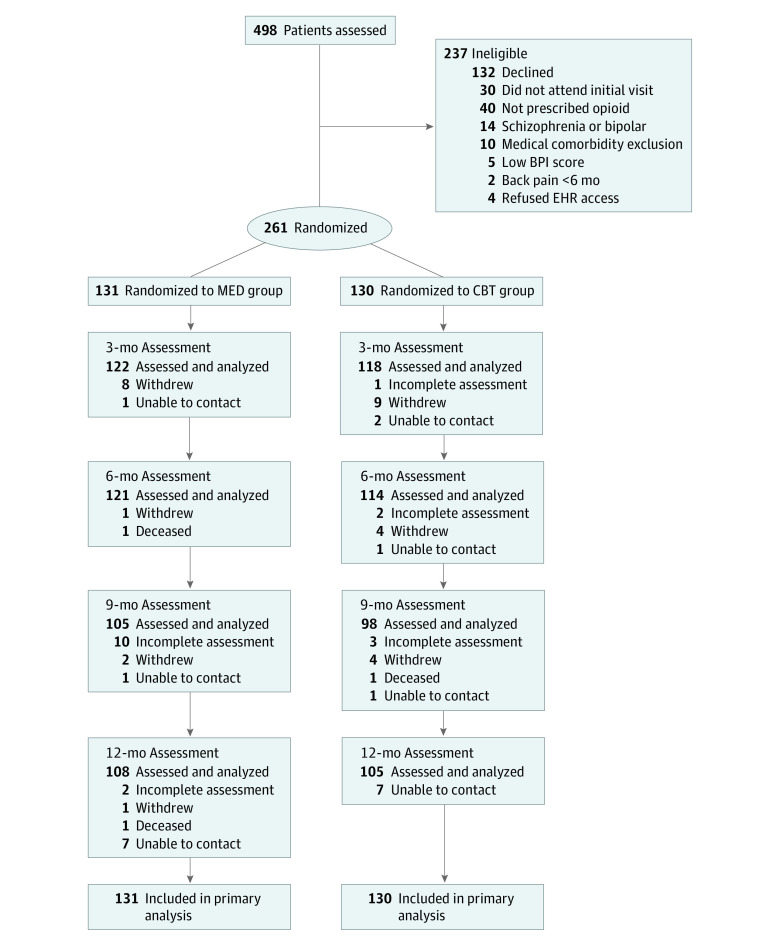
Flow of Participants Through the Care Management for the Effective Use of Opioids Study BPI indicates Brief Pain Inventory; CBT, cognitive behavioral therapy; EHR, electronic health record; and MED, medication optimization.

The 261 CAMEO study participants included 241 men (92.3%) and 20 women (7.7%), with a mean (SD) age of 57.9 (9.5) years. Participants self-reported their race or ethnicity; 54 (20.7%) were Black, 191 (73.2%) were White, and 16 (6.1%) were of another race or ethnicity (including American Indian or Alaska Native; Asian; Hispanic, Spanish, or Latino; and other [write-in response]), did not know their race or ethnicity, or refused to answer. Mean (SD) baseline measure scores were BPI total score of 6.5 (1.7) (moderately severe pain),^22^ RMDQ score of 16.7 (4.4) (moderately severe disability),^38^ pain duration of 22.3 (13.4) years, 3.8 (2.2) medical comorbidities (of 14 conditions screened), PHQ-9 score of 11.2 (6.1) (moderate depression),^30^ and Pain Catastrophizing Scale score of 24.2 (12.0) (60th percentile).^39^ One hundred thirteen patients (43.3%) reported a history of problematic substance use, with a mean (SD) COMM score of 8.8 (6.7) (where a threshold of 9 suggests problematic drug-related behaviors).^28^ Before randomization, participants were asked to rate their optimism about the helpfulness of each intervention on a 10-point scale, with 10 being most optimistic. Optimism about the medication optimization intervention was higher than optimism about CBT (mean score, 5.9 [2.4] vs 4.8 [2.4]; *P* < .001). The participant flow in the CAMEO trial is summarized in Figure 1. Recruitment occurred from September 1, 2011, to December 31, 2014, and follow-up was completed December 31, 2015. Randomization resulted in 131 and 130 participants in the MED and CBT groups, respectively, with similar baseline characteristics (Table 1). Outcome assessments were completed by 240 participants (92.0%) at 3 months, 235 (90.0%) at 6 months, 203 (77.8%) at 9 months, and 213 (81.6%) at 12 months and differed by no more than 4% between groups at any time point. Twenty-nine participants (11.1%) withdrew from the study (12 in the MED group and 17 in the CBT group).

**Table 1.** Baseline Characteristics in Care Management for the Effective Use of Opioids Trial Participants

Characteristic	Participant group^a^	
	MED (n = 131)	CBT (n = 130)
Age, mean (SD), y	57.9 (9.8)	58.0 (9.2)
Sex		
Men	124 (94.7)	117 (90.0)
Women	7 (5.3)	13 (10.0)
Race		
Black	29 (22.1)	25 (19.2)
White	91 (69.5)	100 (76.9)
Other^b^	11 (8.4)	5 (3.8)
Educational level above high school	81 (61.8)	81 (62.3)
Married	64 (48.9)	75 (57.7)
Employment		
Employed	29 (22.1)	25 (19.2)
Retired	32 (24.4)	41 (31.5)
Out of work or unable to work	67 (51.1)	62 (47.7)
Other	3 (2.3)	2 (1.5)
Income adequate^c^	95 (72.5)	92 (70.8)
Disability compensation	100 (76.3)	93 (71.5)
Duration of pain, mean (SD), y	23.0 (13.1)	21.6 (13.7)
Pain treatment history		
Physical therapy	94 (71.8)	90 (69.2)
Orthopedic and/or rheumatology	82 (62.6)	74 (56.9)
Psychiatrist, psychologist, and/or counselor	71 (54.2)	81 (62.3)
Pain clinic	91 (69.5)	74 (56.9)
Chiropractic	61 (46.6)	61 (46.9)
Back surgery	40 (30.5)	33 (25.4)
Pain group or school	34 (26.0)	25 (19.2)
Massage	31 (23.7)	25 (19.2)
Acupuncture	18 (13.7)	15 (11.5)
Confidence in the intervention, mean (SD)^d^		
MED	5.8 (2.5)	6.0 (2.2)
CBT	4.8 (2.3)	4.8 (2.3)
No. of comorbid conditions, mean (SD)^e^	3.8 (2.2)	3.9 (2.1)
Reported substance use within past 3 mo^f^	24 (18.3)	17 (13.1)
Problematic substance use history by report^g^	57 (43.5)	56 (43.1)
Morphine milligram equivalents, median (IQR)	40 (23-69)	45 (30-90)

Abbreviations: CBT, cognitive behavioral therapy; MED, medication optimization.

^a^ Unless otherwise indicated, data are expressed as No. (%) of participants. Percentages have been rounded and may not total 100.

^b^ Includes American Indian or Alaska Native; Asian; Hispanic, Spanish, or Latino; and other (write-in response), do not know, or refused to answer.

^c^ Patients were asked, “When you consider your household income from all sources, would you say that you are comfortable, have just enough to make ends meet, or do not have enough to make ends meet?” Responses of “comfortable” and “just enough” were scored as “adequate.”

^d^ Patients were asked, “How optimistic are you that this study will help improve your back pain if you are in the (MED/CBT) group?” Scores ranged from 0 to 10, with 0 indicating not at all optimistic and 10, extremely optimistic.

^e^ Medical and psychiatric comorbidities were assessed with a 14-item screener assessing for asthma, hypertension, diabetes, arthritis, heart disease, neurological disease, liver disease, kidney disease, cancer, depression, posttraumatic stress disorder, generalized anxiety disorder, traumatic brain injury, and panic attacks.

^f^ Patients were asked if they had used “medicine for pain, sleep, or nerves that was prescribed for another person,” “marijuana,” or “other street drugs (such as cocaine, speed, meth, heroin).” Responses were categorized as “within the past 3 months,” “3 to 12 months ago,” “1 to 10 years ago,” “more than 10 years ago,” and “never.”

^g^ Patients were asked, “In your lifetime, have you ever had a problem with drugs or alcohol?” and “Have you ever received treatment or counseling for a drug or alcohol problem?” A response of “yes” to either question counted as a positive response for this variable.

### Treatment Adherence

In the MED group, 130 participants (99.2%) attended at least 1 session and 119 (90.8%) attended at least 5 sessions, with a mean (SD) of 7.2 (1.9) (median, 8 [IQR, 7-8]) sessions attended. The mean (SD) session length was 24.5 (21.6) minutes. In the CBT group, 123 participants (94.6%) attended at least 1 session and 86 (66.2%) attended at least 5 sessions, with a mean (SD) of 5.7 (2.8) (median, 7 [IQR, 3-8]) sessions attended. Fewer than one-quarter (185 of 802 [23.1%]) of the sessions were completed in person compared with 607 of 802 (75.7%) over the telephone. For 10 of 802 sessions (1.2%), session delivery method was not recorded. The mean (SD) session length was 40.7 (9.4) minutes.

### Primary Outcomes

Improvements in BPI total score were significantly greater in the MED group at 12 months (between-group difference, −0.54 [95% CI, −1.18 to −0.31]; *P* = .04; effect size, 0.31 SD) but not at 6 months (between-group difference, −0.46 [95% CI, −0.94 to 0.11]; *P* = .07; effect size, 0.27 SD), as shown in Table 2. The BPI intensity subscale likewise improved significantly in the MED group compared with the CBT group, both at 12 months (between-group difference, −0.62 [95% CI, −1.19 to −0.16]; *P* = .004; effect size, 0.40 SD) and 6 months (between-group difference, −0.53 [95% CI, −0.95 to −0.03]; *P* = .01; effect size, 0.34 SD). However, between-group BPI interference scores were not significantly different at either 12 months (between-group difference, −0.48 [95% CI, −1.25 to 0.04]; *P* = .09; effect size, 0.24 SD) or 6 months (between-group difference, −0.39 [95% CI, −1.00 to 0.21]; *P* = .14; effect size, 0.19 SD).

**Table 2.** Primary Outcomes in Care Management for the Effective Use of Opioids Trial Participants

Outcome	Score, mean (SD)^a^		Time-specific between-group difference (95% CI)	Within-group score change from baseline, mean (SE)^a^		*P* value^b^
	MED group (n = 131)	CBT group (n = 130)		MED group (n = 131)	CBT group (n = 130)	
BPI total pain score change						
Baseline	6.45 (1.79)	6.49 (1.67)	−0.05 (−0.47 to 0.37)	NA	NA	NA
3 mo	5.41 (2.10)	5.86 (1.91)	−0.45 (−0.09 to 0.07)	−1.04 (0.22)	−0.63 (0.21)	.08
6 mo	5.39 (2.21)	5.85 (1.84)	−0.46 (−0.94 to 0.11)	−1.06 (0.22)	−0.64 (0.21)	.07
9 mo	5.29 (2.25)	5.66 (1.91)	−0.37 (−0.90 to 0.26)	−1.16 (0.23)	−0.83 (0.22)	.18
12 mo	5.31 (2.27)	5.85 (1.98)	−0.54 (−1.18 to −0.31)	−1.14 (0.22)	−0.64 (0.22)	.04
Overall	NA	NA	NA	−1.10 (0.20)	−0.68 (0.19)	.03
BPI intensity score change						
Baseline	6.78 (1.65)	6.76 (1.47)	0.02 (−0.36 to 0.40)	NA	NA	NA
3 mo	5.96 (1.86)	6.24 (1.56)	−0.30 (−0.74 to 0.14)	−0.82 (0.20)	−0.52 (0.19)	.14
6 mo	5.94 (1.95)	6.45 (1.60)	−0.53 (−0.95 to −0.03)	−0.84 (0.20)	−0.31 (0.19)	.01
9 mo	5.84 (1.83)	6.18 (1.69)	−0.36 (−0.82 to 0.16)	−0.94 (0.20)	−0.58 (0.19)	.10
12 mo	5.76 (2.14)	6.36 (1.59)	−0.62 (−1.19 to −0.16)	−1.02 (0.20)	−0.40 (0.19)	.004
Overall	NA	NA	NA	−0.90 (0.17)	−0.45 (0.16)	.006
BPI interference score change						
Baseline	6.34 (2.07)	6.39 (1.99)	−0.06 (−0.55 to 0.44)	NA	NA	NA
3 mo	5.19 (2.38)	5.71 (2.28)	−0.52 (−1.10 to 0.09)	−1.15 (0.26)	−0.68 (0.25)	.08
6 mo	5.17 (2.51)	5.61 (2.15)	−0.39 (−1.00 to 0.21)	−1.17 (0.26)	−0.78 (0.25)	.14
9 mo	5.08 (2.57)	5.47 (2.19)	−0.34 (−0.98 to 0.35)	−1.26 (0.26)	−0.92 (0.25)	.24
12 mo	5.15 (2.46)	5.68 (2.3)	−0.48 (−1.25 to 0.04)	−1.19 (0.26)	−0.71 (0.25)	.09
Overall	NA	NA	NA	−1.19 (0.23)	−0.77 (0.22)	.05

Abbreviations: BPI, Brief Pain Inventory; CBT, cognitive behavioral therapy; MED, medication optimization; NA, not applicable.

^a^ The numbers of participants assessed at follow-up were 239 (121 in the MED group and 118 in the CBT group) at 3 months, 235 (121 in the MED group and 114 in the CBT group) at 6 months, 203 (105 in the MED group and 98 in the CBT group) at 9 months, and 213 (108 in the MED group and 105 in the CBT group) at 12 months.

^b^ Calculated for score change from multivariable mixed-effects models accounting for within-participant correlation for repeated measures and adjusted for baseline outcome measure score as well as baseline depression, sex, and time.

**Figure 2.**
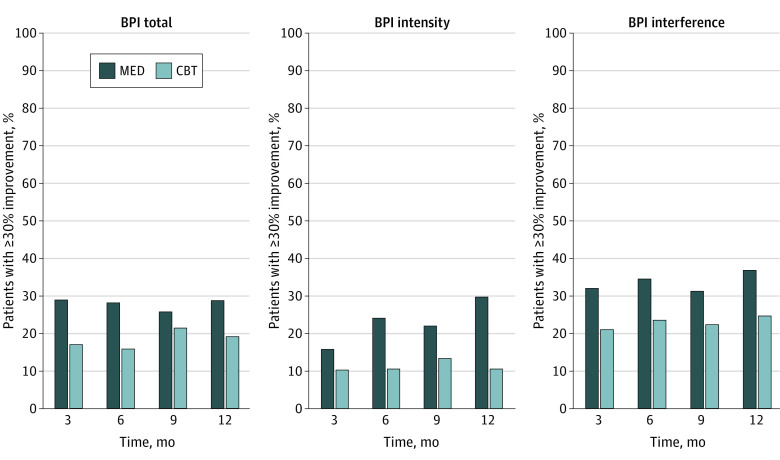
Percentages of Patients Demonstrating at Least 30% Improvement From Baseline Brief Pain Inventory (BPI) Scores CBT indicates cognitive behavioral therapy; MED, medication optimization.

Patients in the MED group were also more likely to have a pain response (ie, ≥30% improvement in pain impact by 12 months) compared with the CBT group (odds ratio [OR] for BPI total score, 1.87 [95% CI, 1.11-3.15; *P* = .02]; OR for BPI intensity score, 2.48 [95% CI, 1.44-4.27; *P* = .001]; and OR for BPI interference score, 1.88 [95% CI, 1.13-3.10; *P* = .01]). The proportion of participants achieving 30% improvement in pain at each time point is shown in Figure 2. The absolute risk reduction (ARR) and number needed to treat (NNT) for the MED compared with the CBT intervention to achieve pain response were as follows: ARR = 12.3% and NNT = 8.1 for BPI total score, ARR = 13.5% and NNT = 7.4 for BPI intensity score, and ARR = 11.0% and NNT = 9.1 for BPI interference score at 6 months; ARR = 9.6% and NNT = 10.4 for BPI total score, ARR = 19.1% and NNT = 5.2 for BPI intensity score, and ARR = 12.2% and NNT = 8.2 for BPI interference score at 12 months.

### Secondary Outcomes

As shown in Table 3, no significant between-group differences were found in secondary outcome measures. Between-group differences at 12 months were −0.98 (95% CI, −2.49 to 0.54; *P* = .22) for pain-related disability (RMDQ), 1.71 (95% CI, −1.79 to 5.21; *P* = .15) for pain catastrophizing, −0.31 (95% CI, −1.82 to 1.21; *P* = .85) for alcohol misuse (Alcohol Use Disorders Identification Test–Concise), −1.37 (95% CI, −3.38 to 0.65; *P* = .12) for opioid misuse (COMM), 0.26 (95% CI, −5.60 to 6.11; *P* = .44) for health-related quality of life (SF-36 General Health), 3.70 (95% CI, −3.96 to 11.37; *P* = .28) for SF-36 Social Functioning, 2.32 (95% CI, −2.01 to 6.66; *P* = .96) for SF-36 Vitality, −0.40 (95% CI, −2.18 to 1.37; *P* = .64) for depression (PHQ-9), and −0.61 (95% CI, −2.00 to 0.78; *P* = .57) for anxiety (GAD-7).

**Table 3.** Secondary Outcomes in Care Management for the Effective Use of Opioids Trial Participants

Outcome	Score, mean (SD)^a^		Time-specific between-group difference (95% CI)	Within-group score change from baseline, mean (SE)^a^		*P* value^b^
	MED group (n = 131)	CBT group (n = 130)		MED group (n = 131)	CBT group (n = 130)	
Roland Morris Disability Questionnaire						
Baseline	16.59 (4.70)	16.72 (4.15)	−0.13 (−1.21 to 0.95)	NA	NA	NA
3 mo	15.55 (5.18)	15.80 (5.20)	−0.24 (−1.57 to 1.08)	−1.02 (0.42)	−0.98 (0.43)	.95
6 mo	15.32 (5.20)	15.34 (5.50)	−0.02 (−1.39 to 1.36)	−1.30 (0.42)	−1.46 (0.43)	.79
9 mo	15.54 (5.53)	15.33 (5.50)	0.22 (−1.31 to 1.74)	−1.15 (0.44)	−1.50 (0.45)	.58
12 mo	15.60 (5.85)	16.58 (5.34)	−0.98 (−2.49 to 0.54)	−0.99 (0.44)	−0.23 (0.44)	.22
Overall	NA	NA	NA	−1.12 (0.34)	−1.04 (0.34)	.88
Pain catastrophizing						
Baseline	23.95 (12.77)	24.44 (11.27)	−0.50 (−3.44 to 2.45)	NA	NA	NA
6 mo	21.64 (13.20)	20.41 (12.54)	1.23 (−2.08 to 4.55)	−2.33 (0.93)	−3.75 (0.95)	.22
12 mo	22.14 (13.84)	20.43 (12.04)	1.71 (−1.79 to 5.21)	−2.00 (0.97)	−3.99 (0.99)	.15
Overall	NA	NA	NA	−2.16 (0.80)	−3.87 (0.82)	.14
AUDIT-C						
Baseline	5.65 (5.51)	5.87 (5.71)	−0.22 (−1.59 to 1.15)	NA	NA	NA
6 mo	5.42 (5.69)	5.75 (5.40)	−0.32 (−1.75 to 1.10)	−0.34 (0.37)	−0.34 (0.38)	.77
12 mo	5.06 (5.58)	5.36 (5.66)	−0.31 (−1.82 to 1.21)	−0.39 (0.39)	−0.50 (0.40)	.85
Overall	NA	NA	NA	−0.37 (0.31)	−0.42 (0.32)	.90
Current opioid misuse measure						
Baseline	8.73 (6.81)	8.87 (6.54)	−0.13 (−1.77 to 1.50)	NA	NA	NA
6 mo	7.29 (6.96)	8.20 (6.85)	−0.91 (−2.69 to 0.86)	−1.52 (0.54)	−0.62 (0.56)	.26
12 mo	7.60 (6.96)	8.96 (7.94)	−1.37 (−3.38 to 0.65)	−0.92 (0.56)	0.19 (0.58)	.12
Overall	NA	NA	NA	−1.22 (0.47)	−0.22 (0.49)	.14
SF-36 General Health						
Baseline	43.66 (20.92)	45.39 (18.64)	−1.73 (−6.56 to 3.10)	NA	NA	NA
6 mo	45.85 (19.89)	45.83 (20.40)	0.01 (−5.17 to 5.19)	1.50 (1.37)	0.23 (1.41)	.52
12 mo	42.70 (23.03)	42.44 (20.17)	0.26 (−5.60 to 6.11)	−1.71 (1.44)	−3.30 (1.46)	.44
Overall	NA	NA	NA	−0.10 (1.16)	−1.53 (1.19)	.39
SF-36 Social Functioning						
Baseline	48.47 (27.21)	49.81 (29.32)	−1.33 (−8.23 to 5.56)	NA	NA	NA
6 mo	55.79 (29.67)	51.10 (27.40)	4.69 (−2.66 to 12.04)	7.42 (2.23)	2.14 (2.29)	.10
12 mo	55.72 (28.49)	52.02 (28.12)	3.70 (−3.96 to 11.37)	6.48 (2.34)	2.88 (2.36)	.28
Overall	NA	NA	NA	6.95 (1.96)	2.51 (1.99)	.11
SF-36 Vitality						
Baseline	38.64 (16.98)	35.08 (16.17)	3.56 (−0.48 to 7.60)	NA	NA	NA
6 mo	40.00 (17.50)	38.33 (17.06)	1.67 (−2.79 to 6.12)	2.37 (1.24)	2.97 (1.27)	.74
12 mo	40.51 (16.28)	38.19 (15.74)	2.32 (−2.01 to 6.66)	2.48 (1.30)	2.57 (1.31)	.96
Overall	NA	NA	NA	2.42 (1.07)	2.77 (1.09)	.82
PHQ-9 Depression						
Baseline^c^	11.18 (6.40)	11.19 (5.82)	−0.00 (−1.50 to 1.49)	NA	NA	NA
3 mo	9.74 (6.33)	9.94 (6.14)	−0.20 (−1.79 to 1.39)	−1.49 (0.46)	−1.19 (0.47)	.65
6 mo	9.74 (6.56)	10.04 (6.12)	−0.31 (−1.94 to 1.32)	−1.55 (0.46)	−1.24 (0.47)	.63
9 mo	9.67 (6.41)	9.84 (5.68)	−0.17 (−1.85 to 1.51)	−1.75 (0.49)	−1.40 (0.50)	.62
12 mo	10.13 (6.95)	10.53 (6.21)	−0.40 (−2.18 to 1.37)	−1.04 (0.48)	−0.72 (0.49)	.64
Overall	NA	NA	NA	−1.46 (0.36)	−1.14 (0.37)	.54
GAD-7 Anxiety						
Baseline^d^	6.68 (5.13)	6.81 (4.63)	−0.13 (−1.33 to 1.06)	NA	NA	NA
3 mo	6.65 (4.50)	6.08 (4.57)	0.57 (−0.65 to 1.79)	−0.16 (0.33)	−0.67 (0.34)	.28
6 mo	6.00 (5.23)	6.22 (4.88)	−0.22 (−1.52 to 1.08)	−0.78 (0.33)	−0.62 (0.34)	.74
9 mo	6.30 (4.92)	5.92 (4.83)	0.39 (−0.96 to 1.74)	−0.49 (0.35)	−1.09 (0.36)	.23
12 mo	6.41 (5.30)	7.02 (4.98)	−0.61 (−2.00 to 0.78)	−0.17 (0.35)	0.11 (0.36)	.57
Overall	NA	NA	NA	−0.40 (0.25)	−0.57 (0.25)	.63

Abbreviations: AUDIT-C, Alcohol Use Disorders Identification Test–Concise; CBT, cognitive behavioral therapy; GAD-7, Generalized Anxiety Disorder 7-item scale; MED, medication optimization; NA, not applicable; PHQ-9, Patient Health Questionnaire 9-item Depression scale; SF-36, 36-item Short Form Health Survey.

^a^ The numbers of participants assessed at follow-up were 239 (121 in the MED group and 118 in the CBT group) at 3 months, 235 (121 in the MED group and 114 in the CBT group) at 6 months, 203 (105 in the MED group and 98 in the CBT group) at 9 months, and 213 (108 in the MED group and 105 in the CBT group) at 12 months.

^b^ Calculated for score change from multivariable mixed-effects models accounting for within-participant correlation for repeated measures and adjusted for baseline outcome measure score.

^c^ Sixty-seven participants (51.1%) in the MED group and 74 participants (56.9%) in the CBT group had at least moderate depression (PHQ-9 Depression score ≥10) at baseline.

^d^ Twenty-nine participants (22.1%) in the MED group and 39 participants (30.0%) in the CBT group had at least moderate anxiety (GAD-7 score ≥10) at baseline.

### Opioid Dose and Nonopioid Analgesic Use

The baseline distribution of daily prescribed opioid dose was highly skewed (4.4), with a range of 8 to 900 MMEs (eTable 2 in Supplement 2). Median daily opioid dose was 40 (IQR, 30-90) MMEs. Although mean daily MMEs trended lower in the MED group, the between-group difference was not statistically significant at 6 months (61.18 [75.22] MMEs in the MED group vs 72.73 [96.81] MMEs in the CBT group; mean difference, −11.55 [95% CI, −32.73 to 9.62]; *P* = .28) or at 12 months (54.14 [73.29] MMEs in the MED group vs 66.67 [96.12] MMEs in the CBT group; mean difference, −12.53 [95% CI, −33.41 to 8.35]; *P* = .24). For the total number of nonopioid analgesics prescribed at baseline, distribution was positively skewed (1.9) (eTable 3 in Supplement 2). The mean number of nonopioid analgesics prescribed was significantly higher in the MED group compared with the CBT group at 6 months (1.73 [1.27] in the MED group vs 0.75 [0.84] in the CBT group; mean difference, 1.47 [95% CI, 1.192-1.74]; *P* < .001) and at 12 months (1.31 [1.10] in the MED group vs 0.77 [0.95] in the CBT group; mean difference, 0.55 [95% CI, 0.30-0.79]; *P* < .001). These medication dose findings are exploratory, given multiple post hoc analyses.

### Adverse Events

We expected unbalanced adverse event reporting owing to systematic adverse effect assessment by NCMs in the MED group. Serious adverse events were recorded in 8 participants in the MED group (2 deaths, 1 atrial fibrillation, 2 small-bowel obstructions, 2 urinary tract infections, and 1 knee replacement) and 2 in the CBT group (1 death and 1 knee replacement). Ten other adverse events were reported in the MED group compared with 2 in the CBT group.

## Discussion

The CAMEO randomized clinical trial yielded several key findings. First, the collaborative care medication optimization approach was significantly more effective at improving BPI total score (primary outcome) than the CBT intervention, although the differences were modest and unlikely to be clinically meaningful. Second, patients in the MED group were nearly twice as likely (OR, 1.87) to achieve at least a 30% improvement in their BPI total score during the 12-month study. Third, no between-group differences were found in secondary outcomes.

Without a usual care comparator group, a precise effect size for the CBT intervention cannot be calculated, although the magnitude of change in pain scores is consistent with the modest effect sizes reported in prior CBT for pain trials.^40^ DeBar et al^41^ recently compared a primary care–based CBT intervention with usual care in patients receiving long-term opioid therapy for chronic pain and found effect sizes of 0.28 at 3 months and 0.21 at 12 months. The CBT intervention in the CAMEO trial was more robust than the CBT delivered in the previous ESCAPE (Evaluation of Stepped Care for Chronic Pain) stepped-care trial,^15^ with the addition of an in-person option and 2 additional sessions. Adherence to the CBT intervention in the CAMEO trial was comparable with that in other trials of CBT for chronic pain.^40,41,42^

There are several potential reasons that CBT was less effective than medication management and analgesic optimization in the present study. First, participants were more optimistic about medication management than CBT, signaling potential expectancy bias. Although we did not include this optimism measure in our primary analysis, we plan to conduct a secondary analysis to explore this factor further. Second, although baseline pain catastrophizing scores were above average (mean, 24 [60th percentile]), this cohort did not, on average, meet the clinically meaningful threshold score of 30.^39^ Subthreshold catastrophizing scores suggest that CBT may not be an ideal modality in this cohort. Third, the CBT group had a higher rate of participants who withdrew (13.1% vs 9.2%) or did not complete at least 5 intervention sessions (33.8% vs 9.2%) compared with the MED group. Fourth, the complexity of participants may have contributed to treatment refractoriness. In this sample, the mean pain duration was 22.3 (13.4) years, 193 (73.9%) were receiving disability, and 73 (28.0%) had previous back surgery. This sample also reported higher medical comorbidity compared with previous trials (mean [SD] comorbid conditions, 3.8 [2.2] in the CAMEO trial compared with a range of 1 [1] to 2.7 [1.5] in 4 prior trials of collaborative care for pain).^13,14,15,43^ This complexity may also be responsible for the lack of meaningful improvement on secondary measures for either treatment group.

More adverse events were observed in the MED group compared with the CBT group. It is possible that active medication adjustments and related adverse effects could have contributed to more adverse events. However, it is also possible that this difference is accounted for by systematic assessment of adverse effects by the NCMs.

We hypothesized that there would be a clinically meaningful difference in BPI pain scores between study treatments at 6 and 12 months. Contrary to our hypothesis, we found that the between-intervention difference was only significant at 12 months. This finding, along with the lack of between-group differences in secondary measures, suggests that although we detected a between-group difference, this difference may not be clinically meaningful. Our findings show that the NCM-led medication optimization intervention improved pain more than CBT. However, this difference did not meet the clinically meaningful threshold at both time points tested that would support a claim of superiority.

### Strengths and Limitations

The CAMEO trial has notable limitations and strengths. One limitation is that the trial recruited veterans with CLBP receiving long-term opioid therapy from Veterans Affairs clinics, requiring caution when generalizing results to nonveteran populations; however, this narrowly defined population is also a strength because pain improved in both groups despite the lengthy duration of pain, substantial comorbidity, and severe disability. The 2-group design of CAMEO is another limitation because it is not possible to adequately judge the effect of the CBT intervention without a usual care group. However, a 3-group design would not have been feasible within our recruitment catchment. Despite maximizing our recruitment window within the study funding period, we had difficulty achieving our recruitment target in this 2-group study. Our power to detect the 0.3-SD difference we found during the 12-month study period was only 62%. Replicating these findings will require much larger sample sizes, especially if a 3-group design is pursued. Difficult recruitment coupled with extended data extraction and analysis of secondary outcomes contributed to the delayed publication of this report. However, with the current national emphasis on minimizing opioid prescriptions, this comparison of strategies to improve pain in veterans prescribed opioids remains highly relevant.

## Conclusions

The CAMEO randomized clinical trial is, to our knowledge, the first comparative effectiveness study to compare collaborative care medication optimization with CBT in patients with CLBP who were prescribed opioids. Our results indicate that a collaborative care medication optimization approach has a statistically significant but clinically modest benefit when compared with CBT for 12 months. This finding suggests that both pharmacological and behavioral approaches are reasonable options for chronic pain.

## Notes

Supplement 1.
Trial Protocol

Supplement 2. **eTable 1.** List of Analgesics Assessed on Medical Record Review **eTable 2.** Prescribed Opioid Morphine Milligram Equivalent (MME) Data **eTable 3.** Number of Prescribed Analgesics Comparison

Supplement 3.
Data Sharing Statement
