# Supplementary material for: Effect of Medication Optimization vs Cognitive Behavioral Therapy Among US Veterans With Chronic Low Back Pain Receiving Long-term Opioid Therapy: A Randomized Clinical Trial
Source: JAMA Netw Open. 2022 Nov 17;5(11):e2242533. doi: 10.1001/jamanetworkopen.2022.42533 (PMC9672973; doi:10.1001/jamanetworkopen.2022.42533)
Supplement: Supplement 1. — Trial Protocol [file jamanetwopen-e2242533-s001.pdf]

CAMEO PROTOCOL

A. RESEARCH OBJECTIVES

Chronic pain is a major public health problem. More than 70 million Americans suffer from chronic pain and 50 million of them are disabled by pain.[1] Chronic pain affects 40%-70% of veterans[2] and is a leading cause of disability, resulting in substantial negative impact on millions of veterans' lives.[2] Chronic pain is frequently accompanied by psychiatric comorbidity that adds to patient suffering and complicates treatment.[3] Chronic pain costs more than $100 billion per year in medical expenses, lost wages due to disability, and other costs.[4] Musculoskeletal pain is especially common, accounting for two-thirds of all primary care visits for pain,[5] and chronic low back pain (CLBP) is the most prevalent, disabling, and costly.

Many options are available to treat CLBP, yet management is difficult because of the lack of consensus to guide clinician decisions. Analgesic medications remain the first line of treatment, but providers often do not use the entire array of analgesics that have been shown in clinical trials to be efficacious for CLBP. For non-pharmacological treatments, the strongest trial evidence is for those which use cognitive or behavioral approaches. Despite this evidence, primary care settings have not routinely implemented non-pharmacological treatments for CLBP because of time constraints, lack of provider knowledge, and limited personnel to deliver non-pharmacological treatments. However, the recent integration of psychologists into VA primary care settings increases the feasibility of administering non-pharmacological interventions. While multidisciplinary pain clinics produce the best outcomes using both pharmacological and non-pharmacological treatments,[6] the availability of such clinics is limited. Even if more referral services were available, the enormous burden of CLBP among veterans requires that most management still needs to occur in the primary care setting.

Use of opioid analgesics has increased both outside and within the VA for many pain conditions, including CLBP. While some pain experts view this trend as evidence of improved pain treatment, others have equated this practice to "flying blind,"[7] given the paucity of trials evaluating the effectiveness and safety of opioids. Many patients continue to experience severe, disabling pain despite opioid treatment; others report intolerable side effects from opioids. Primary care providers often struggle with opioid treatment decisions and worry about fostering prescription drug abuse and addiction. Given these controversies, struggles, and lack of convincing data for opioid use, research to compare pharmacological and non-pharmacological treatments to improve the management of CLBP, especially for veterans on long-term opioid therapy, is urgently needed.

Our long-term research objective is to develop, test, and implement novel treatments and care delivery models that address barriers to effective pain management and that can be practicably applied in VA primary care settings. The **CA**re **M**anagement for the **E**ffective use of **O**pioids **(CAMEO)** trial is a 2-arm randomized clinical trial to compare the effectiveness of pharmacological vs. behavioral approaches for CLBP.

Our study sample will include 300 veterans with moderate to severe CLBP despite long-term opioid therapy. Patients from five primary care clinics at the Roudebush VA Medical Center and *three community based outpatient clinics* will be recruited to participate in CAMEO and randomized to one of two treatment arms. The pharmacological arm will involve guideline-concordant opioid management coupled with algorithm-based co-analgesic treatment **(PHARM)**. Patients in the behavioral arm **(BEH)** will receive pain self-management/coping skills training. The trial will last 12-months and all participants will undergo comprehensive outcome assessments at baseline, 3, 6, 9, and 12 months.

Study Aims: Among veterans with chronic low back pain refractory to long-term opioid therapy

1) **To compare the interventions' (PHARM vs. BEH) effects on pain intensity and function at 6 months (primary end point) and 12 months (sustained effect)**
2) **To compare the interventions' effects (PHARM vs. BEH) on other relevant outcomes**
    - Patient global impression of change
    - Health-related quality of life
    - Opioid dose
3) **To compare the cost-effectiveness of the interventions**

The trial is powered to detect a clinically significant between-group treatment effect. In addition to determining whether there is differential clinical effectiveness between the two types of treatments, the relative cost-effectiveness of the interventions will be determined. Because of clinical equipoise, it is not clear which

treatment approach will produce the most meaningful differences in outcomes. Therefore, the null hypothesis of no clinically significant difference between the two active treatment arms would be satisfied given a treatment effect size < 0.3 standard deviation. We expect both active treatment arms to significantly improve pain intensity, function, and other relevant outcomes at 6 and 12-months compared to baseline.

B. BACKGROUND

B1. Chronic pain is a common, costly, and frequent cause of morbidity

Pain is the most common physical symptom reported in both the general population and in primary care,[8;9] accounting for one-fifth of all clinic visits – over 100 million outpatient visits in the U.S. each year.[5] Further, pain takes an enormous toll on the US economy, with lost productive time from common pain conditions costing an estimated $61.2 billion per year.[10] An estimated 1.3 billion person-days of work lost each year are due to back pain alone.[11] Pain adversely influences almost every aspect of a person's life and is frequently associated with depression and other psychiatric conditions. Studies have documented inadequate pain management in a variety of settings.[12;13] Notably, it has been estimated that 4 out of every 10 people with moderate to severe pain do not get adequate relief.[14]

B2. Low back pain is the most prevalent, disabling, and costly pain condition

Low back pain—the most common type of pain seen in primary care— ranks second only to the common cold as a reason for office visits.[15] In a systematic review of 56 studies examining the population prevalence for low back pain, the point prevalence ranged from 12% to 33%, 1-year prevalence ranged from 22% to 65%, and lifetime prevalence ranged from 11% to 84%.[16] Low back pain remains a leading cause of worker disability in the U.S., with costs of over $400 million in compensation claims alone. The direct medical costs and costs of lost productivity from low back pain are estimated at $25 billion and $28 billion per year, respectively.[17] Factors associated with the development of chronic disability due to low back pain include psychological distress, presence of other types of chronic pain, job dissatisfaction or stress, and disputes over compensation.

B3. Challenges to implement effective treatments for chronic low back pain in primary care

Recent guidelines highlight the wide range of treatment options for the management of chronic low back pain.[18;19] Therapies that have proven efficacy in CLBP include simple analgesics (NSAIDs), tricyclic antidepressants, exercise therapy, and psychological therapies. Medications are the most common treatment, but often provide suboptimal pain relief when used as mono-therapy. Trial evidence for non-pharmacological treatments is strongest for those using cognitive or behavioral approaches.[20;21] For example, pain self-management programs have proven efficacious in trials of low back pain.[22] Despite this evidence, primary care settings have not routinely implemented non-pharmacological treatments for CLBP. Barriers to implementation are largely administrative and systemic and include time constraints, lack of knowledge in behavioral and self-management strategies, and limited availability of specialists to deliver behavioral treatments.

B4. Prescription opioid use and abuse are rising in parallel with each other

In the last 20 years, opioid therapy has expanded beyond cancer pain into widespread use for all types of moderate to severe acute and chronic non-cancer pain. Despite evidence gaps, primary care providers (PCPs) are prescribing opioids with greater frequency.[23] Between 1990 and 1996 the medical use of the opioids fentanyl, morphine, and oxycodone increased 1168%, 59%, and 23%, respectively.[24] More recently, Zerzan et al.[25] found that opioid use increased 309% in a 7-year period and that dispensing of opioids in Medicaid programs increased at almost twice the rate of non-pain related medications. According to our hospital's pharmacy leadership, RVAMC prescribes more opioids than any other VA Medical Center. Data shows that there were over 67,000 prescriptions for opioids in FY 2009, equating to a total pharmacy cost of $3.2 million

While opioid prescriptions are rising, abuse (i.e., use of an opioid in a manner other than how it is indicated or prescribed) is escalating at a commensurate rate.[26] Abuse of prescription opioids rose 71% between 1997 and 2002,[27] and a single-site VA study found that 24% of patients receiving opioids had misuse documented in their charts.[26] More alarmingly, recent reports indicate that opioid-related deaths are also on the rise.[28] Between 1999 and 2004, deaths from methadone poisoning rose 390%, compared to 54% for drugs overall.[29]

B5. The safe and effective use of opioids in primary care is challenging

PCPs manage the vast majority of patients with chronic pain, yet many have received little training in pain or addiction medicine. Further, PCPs face a dearth of evidence to guide prescribing and monitoring, and lack

resources and system supports to facilitate high quality pain and opioid management.[30] PCPs are often not well-versed in the nuances of using opioids, or in tools such as treatment agreements ("contracts") and urine drug tests to monitor misuse (i.e. use of opioids in a manner other than indicated or prescribed).

Currently, most primary care settings are not equipped to facilitate a comprehensive evaluation of patients with chronic pain for treatment response, side effects, adherence, and misuse.[31] Time constraints, limited resources, and competing demands may interfere with the effective pain management. Dobscha et al.[32] found that three-quarters of VA PCPs viewed treating chronic pain as a "major" source of frustration, largely because of perceived inability to offer optimal treatments and their view that system support is inadequate for managing the complexities of chronic pain (i.e., disability and associated psychiatric disorders). We conducted PCP interviews here at RVAMC that corroborate Dobscha's findings.[33] PCPs often face the dilemma of balancing the pain-relief potential of opioids for patients who need them against the reality that some may misuse them.

B6. Other treatment options are needed in primary care when opioids fail to relieve veterans' pain

While opioids have substantially and safely improved the quality of life for many patients disabled by pain,[34] their long-term use remains controversial for several reasons. First, the long-term efficacy of opioids is unclear, especially for CLBP.[35] Second, opioids are associated with problematic side effects such as constipation, sedation, nausea, dry mouth, and itching. Third, opioids may lead to adverse physiologic effects such as hyperalgesia[36] and hypogonadism[37] and have potential for misuse, which may occur more frequently than previously thought. Fourth, opioids provide significant relief for only a minority of patients.[38]

The lack of relief from opioids is frustrating to patients who may feel like they have exhausted all options in their pain treatment. This is also frustrating for PCPs concerned about opioid dose escalation and lack of additional treatment options. Guidelines and reviews[6] suggest that patients with refractory pain could benefit from multidisciplinary and multimodal pain treatment, but such intensive treatment is often not available. Patients and PCPs need additional treatment options, delivery models, or system supports that can be practicably applied in a busy VA primary care setting.

B7. Care management holds promise as a delivery model to improve VA pain care

Studies of nurse care management for pain appear promising as a care delivery model to support PCPs. Lamb et al.[39] found that a nurse-led case management program for methadone treatment improved safety and patient satisfaction. Our SCAMP (Stepped-Care for Affective Disorders and Musculoskeletal Pain) trial, published last year in *JAMA*,[40] tested a stepped care approach for primary care patients with comorbid depression and chronic musculoskeletal pain. All aspects of the intervention (antidepressants combined with a pain self-management program) were delivered by nurse care managers. At 12-months, the intervention showed large reductions in depression severity and moderate benefits for pain severity and function.[40]

The VA's Patient Centered Medical Home Initiative and its focus on an interdisciplinary, team-based model of care delivery to facilitate partnerships among veterans, PCPs, and other health care professionals such as nurse care managers and clinical psychologists is relevant to chronic pain management in general and CAMEO specifically. By comparing two different interventions (pharmacological vs. behavioral) and delivery models (nurse care management vs. primary care-mental health integration) in CAMEO, findings will inform the evolution of VA's Patient Centered Medical Home.

B8. Non-pharmacological treatments have been shown to be effective for chronic pain

For patients with chronic pain, self-management involves a combination of treatment adherence, behavioral change, adapting life roles, managing negative emotions, and coping skills. A systematic review by Newman et al.[41] found strong clinical trial evidence that self-management programs are effective for both low back pain and osteoarthritis, with possible secondary benefits in reducing psychological distress.[22] Furthermore, back pain outcomes may be more dependent on effective self-management than on other treatment approaches.[42] Lorig's Arthritis Self-Management Program is the most commonly cited program and has consistently demonstrated effectiveness in improving and maintaining health outcomes and reducing health care utilization among patients with arthritis and various rheumatic conditions,[43] including low back pain.[44] The program focuses on improving perceived ability through action plans, feedback, emotional management, and problem solving strategies. It may be effectively administered by trained individuals in group or individual settings.

Cognitive-behavior therapy (CBT) is the most evidence-based psychological alternative to traditional medical approaches to CLBP management. Evidence from numerous trials supports CBT for a variety of pain

conditions,[45] including CLBP.[20] CBT for chronic pain is informed by social learning theory that hypothesizes that patients' idiosyncratic beliefs, attitudes, and coping resources play a central role in determining their experience of pain.[46] Accordingly, patients' maladaptive appraisals directly contribute to the persistence of back pain and pain-related disability. CBT for chronic low back pain has evolved as a coping skills education and training approach designed to promote the acquisition and sustained use of a range of adaptive behavioral (e.g., activity pacing, behavioral goal setting) and cognitive (e.g., cognitive reframing, mental relaxation) pain coping skills to promote a perspective of personal control and mastery related to the experience of chronic pain.

B9. Our prior pain research that informs this CAMEO trial

We have performed considerable preliminary work that informs this trial:

1) Pain as the 5th vital sign in veterans. We retrospectively compared opioid prescribing by PCPs before and after implementation of the "Pain as the 5th vital sign" and found that opioid prescriptions significantly increased after implementation. Furthermore, higher pain scores were significantly related to the likelihood of an opioid prescription, suggesting that PCPs tailored their analgesic prescribing to their patients' pain severity.

2) SCAMP trial – quantitative results. Our SCAMP trial (Kroenke, PI) which tested antidepressants combined with a pain self-management program delivered by nurse care managers, showed the intervention group (n = 123) experienced large improvements in depression severity (effect size = 1.1) as compared to usual care patients (n = 127) and moderate improvements in pain severity (effect size = 0.5).[40] While the intervention was effective, the moderate decrease in pain suggests a need for improved analgesic management--a desire repeatedly expressed by SCAMP patients.

3) SCAMP trial – qualitative results. Focus groups (Bair, PI) of SCAMP participants (one-third veterans) identified support from the nurse care managers as one of the most beneficial aspects of the trial.[47] Further, in identifying barriers and facilitators to their use of pain self-management,[48] patient perspectives on opioids emerged, revealing that frustrations are felt by physicians and patients alike.

    (1) "To get medicine, to get pain medicine out of them is like fighting Mohammed Ali and Mike Tyson, you know…you have to be on your, practically on your deathbed to get a pain pill. And they don't understand that. They think I'm addicted. It's not that." (2) And, I felt like a druggie and, I mean, I woke up and I was like, "Oh, God."

4) Clinical reminders for veterans with severe pain. The CRAFT study (Bair, PI) a HSR&D funded project, revealed PCPs' challenges faced related to opioid prescribing. For example:

    (1) "They end up at the VA, and they have already been on narcotics for many years. You have a bad feeling about what is going on here, but by that point, it is kind of like the horse is out of the barn" (2) "I have several doctors in my clinic who I really feel like they spend one nanosecond in the room with the patient and write the narcotics and walk out. Because that is the easy way out. And, if you want to actually try to deal with it and deal with the complexities of it, that takes a lot of time. It takes a lot of attention, and too many doctors don't deal with it."

5) Opioid prescribing and renewals for veterans with chronic pain This pilot study (Krebs, PI) characterized practice gaps for therapeutic monitoring of long-term opioids in primary care. Chart reviews of 123 patients found: (1) 80% had an indication for opioids documented; (2) 84.5% had a pain assessment documented; (3) 30% had adherence assessed; (4) 21% had adverse effects assessed; (5) 15% had a urine drug screen within 12 months; and (6) 14% had an opioid agreement in the medical record. These findings suggest that current opioid monitoring practices fall well short of VA/DoD opioid therapy guidelines.[49]

6) ESCAPE Trial (Bair, PI)—The VA RR&D-funded ESCAPE trial is an ongoing RCT designed to test a stepped care intervention for OIF/OEF veterans with musculoskeletal pain of the spine (low back, neck) and extremities (legs, knees, hips, and shoulders). The intervention involves treatment with analgesics combined with pain self-management skills in step 1. Patients who do not improve at 3-months move on to step 2-- cognitive behavioral therapy. We started patient recruitment in January 2007 and plan to complete enrollment by September 2010.

7) <u>Other current VA pain research among our CAMEO team</u>. Dr. Krebs has a VA HSR&D CDA which focuses on improving opioid prescribing in veterans with chronic pain.

Table 1 below summarizes how CAMEO differs from our completed SCAMP trial and ongoing ESCAPE and SCOPE trials. (Key information and differences in trial characteristics are **bolded**)

**Table 1. Comparisons across proposed, completed, and ongoing trials**				
Characteristics	**CAMEO**	**SCAMP**	**ESCAPE**	**SCOPE**
Status	Proposed (6/10)	**Completed**	Active until (9/10)	Started (10/09)
Patients, N	**300**	250	240	250
% Veterans	100%	40%	**100% OIF/OEF**	100%
Clinic setting	Primary care	Primary care	**Post-deployment** Primary Care	Primary care
Study site(s)	RVAMC **23CBOCs***	Indiana Univ. and RVAMC	RVAMC	RVAMC
Pain condition(s)	**Low back pain**	Knee, hip, and low back pain	Spine and extremity pain	Musculoskeletal pain
Pain severity	**Pain refractory to opioid therapy**	Mod-severe	Mod-severe	Mod-severe
Intervention components and characteristics				
Pharmacological	**Opioid changes**/ Coanalgesics	Anti-depressants	Coanalgesics	Analgesics + anti-depressants
Behavioral	Pain self-management	**+** Pain self-management	**+** Pain self-management	None
Psychological	**+** **Pain coping skills**	None	**+** **Cognitive therapy**	None
Primary outcome	Pain	**Depression/pain**	Pain	Pain
Cost analysis	**Yes**	No	No	No
Control arm	**Head-to-head active comparator**	Usual care	Usual care	Usual care

* Community Based Outpatient Clinics

B10. Studies from other research teams that inform the CAMEO trial

Dr. Jodie Trafton's ATHENA-Opioid Therapy project informs and complements CAMEO—both studies support PCPs in the safe and effective use of opioids. Dr. Trafton and her team designed and evaluated an automated decision support system (ATHENA) that overlays CPRS (Computerized Patient Record System) and provides PCPs with customized recommendations based on VA/DoD opioid guidelines at the point-of-care. Pilot testing demonstrated that PCPs rated the system as usable and clinically relevant. Preliminary data suggested that the system encouraged safe opioid prescribing.[50] However, PCPs did not use the system in clinical practice as much as expected for two reasons: 1) lack of time to implement ATHENA recommendations; and 2) the recommendations lacked specificity to guide clinical decisions. Furthermore, Dr. Trafton has faced barriers to implementation of the ATHENA-Opioid Therapy because of VA informatics software policies and restrictions.

Dr. Steve Dobscha et al tested a collaborative care model for veterans with chronic pain in a VA HSR&D-funded trial.[51] "Assistance with Pain Treatment (APT)" used a clinical psychologist to deliver the multi-component intervention that included: clinician education, patient assessment, education and activation, symptom monitoring, feedback and recommendations to clinicians, and facilitation of specialty care. Compared to usual care, patients in the collaborative care intervention showed greater improvements in pain-related disability and pain interference. Dobscha et al. concluded that the intervention resulted in modest but statistically significant improvements in pain and mental health outcomes. CAMEO differs from APT in a number of respects, including: a) comparison between two active treatment arms rather than usual care; b) a more structured analgesic algorithm and explicit decision rules for stepping up pharmacological management; c) focus on veterans with moderate to severe CLBP despite opioid therapy; d) and use of a structured behavioral intervention that involves pain self-management/coping skills training. Thus, CAMEO and APT will complement one another, providing key advances to deal with the enormous burden of pain in primary care.

C1. SIGNIFICANCE AND RELEVANCE TO VHA PATIENT CARE MISSION

Pain is a critical health problem among veterans. Chronic pain affects 40%-70% of veterans[2] and is a leading cause of disability, with substantial negative impact on millions of veterans' lives. Pain was the most frequently reported symptom in Persian Gulf War veterans [52] and is expected to be even more prevalent in the current cohort of veterans.[53] Of all musculoskeletal pain conditions, chronic low back pain is the most common. Sinott and Wagner[54] found that among veterans receiving VA care, those diagnosed with low back pain increased steadily from 2000 to 2007 at a rate of 4.8% annually--a rate higher than that for either diabetes or hypertension. CLBP is the most common cause of long-term disability, and the related costs in lost productivity are staggering. The VA Health Economics Research Center (HERC) documented the high costs of care for veterans with CLBP--estimated at more than $2 billion in 1999.[55]

The VA has pioneered innovative organizational efforts, such as "Pain as the 5th Vital Sign" initiative and the VHA National Pain Management Strategy in an effort to address pain among veterans. The VHA National Pain Management Strategy was initiated in 1998, and established pain management as a national priority. The overall objective of the national strategy is to develop a comprehensive, multicultural, integrated, system-wide approach to pain management that reduces pain and suffering for veterans experiencing acute and chronic pain associated with a wide range of conditions, including terminal illness. Central to this objective is to assure access to an interdisciplinary approach to pain care across VA facilities. Comparing the effectiveness of pharmacological and behavioral approaches to treat CLBP is critical in meeting strategy objectives.

In October 2009, the VHA Pain Management Directive 2009-053 was released. This directive outlines the VA's stepped-care model of pain treatment, which provides for management of most pain conditions in the primary care setting. Stepped care is a strategy to provide a continuum of effective treatment of patients from acute pain to chronic pain. "Step One" is centered in primary care and requires the development of a competent PCP workforce to manage common pain conditions. To accomplish this, primary care requires the availability of system supports (nurse care management), family and patient education programs, collaboration with integrative mental health-primary care teams, and post-deployment programs. These efforts are supported by timely access to specialty consultation from pain medicine, mental health, physical medicine and rehabilitation, and care coordination for advanced diagnostic and medical management. It is in this context that the CAMEO trial is proposed. Despite the rising prevalence and negative impact of CLBP, few intervention studies have addressed this condition in the primary care setting.

While numerous treatments are available for the management of CLBP, their effectiveness has not been demonstrated convincingly, and consequently, treatments vary widely. Recent evidence-based guidelines published by the American Pain Society and American College of Physicians[18;19] highlight the wide range of approaches and lack of consensus for management. Analgesic medications remain the most common treatment for CLBP. However, while analgesics are the first line of therapy, clinicians do not use the entire array of analgesics shown in clinical trials to be efficacious, and monitoring of pain outcomes and appropriate adjustments (e.g., maximizing doses or switching medications) is often suboptimal in clinical practice.[56] Analgesics with evidence of benefit in low back pain include acetaminophen, NSAIDs, tramadol, gabapentin, skeletal muscle relaxants, tricyclic antidepressants, and serotonin-norepinephrine re-uptake inhibitors.[18]

The use of opioids for the long-term management of CLBP is increasing. Despite this trend the benefits and risks of opioids remain unclear. A 2007 Cochrane Review [57] found opioids, particularly tramadol, were more effective than placebo for pain relief and improved function. However, side effects were common. Further, only 4 trials were reviewed and they were all fraught with methodological flaws, including lack of generalizability, poor descriptions of study populations, poor intention to treat analysis, and limited interpretation of functional improvement. The authors concluded that high quality studies more closely simulate clinical practice are needed to evaluate the risks and benefits of opioids for low back pain. In sum, though systematic reviews and meta-analyses of opioids for various chronic conditions have shown moderate benefits, the evidence to support the use of opioids, specifically for CLBP, is sparse and inconclusive.[35;57]

Given the rising prevalence of CLBP among veterans, the modest effectiveness of current treatments, and the burden chronic pain places on veterans and their PCPs, our research proposal is significant in several regards. *First*, the CAMEO trial directly addresses a high priority area for the VA and is well aligned with the VHA Pain Management Strategy, recently published VHA Pain Directive, VA Primary Care-Mental Health Integration, and VA's Patient Centered Medical Home Initiative. *Second*, our trial will provide information vital to begin filling an evidence vacuum regarding comparative effectiveness of treatments for chronic pain, especially in the primary

care setting. *Third*, opioid prescribing is on the rise within and outside the VA without data to support this practice. The study interventions being tested have the potential to support or challenge this practice trend. *Fourth*, CAMEO will extend our current understanding of pharmacological and behavioral approaches. *Fifth*, the economic evaluation will provide useful information to VA administrators and managers about the short-term budget implications of implementing each of the interventions

D. RESEARCH DESIGN AND METHODS

D1. Conceptual Model for CAMEO Trial

The management of chronic pain is complex. As a result, specialists such as orthopedists, anesthesiologists, rheumatologists, and neurologists, are often consulted. This common practice of referring patients with chronic pain to specialists with a relatively narrow focus compromises the effort to address whole-person care which employs a biopsychosocial approach-- an approach that seems best suited for the primary care setting. The biopsychosocial model posits that the causes and outcomes of many illnesses often involve the interaction of physical and physiological factors, psychological traits and states, and social-environmental factors. Effective pain management accounts for these factors. The biopsychosocial model **(Figure 1)** is the most widely accepted conceptual model in chronic pain management.[58] Comprehensive chronic pain management based on the biopsychosocial model of pain generation and perception improves outcomes.[59]

Figure 1: Biopsychosocial Model

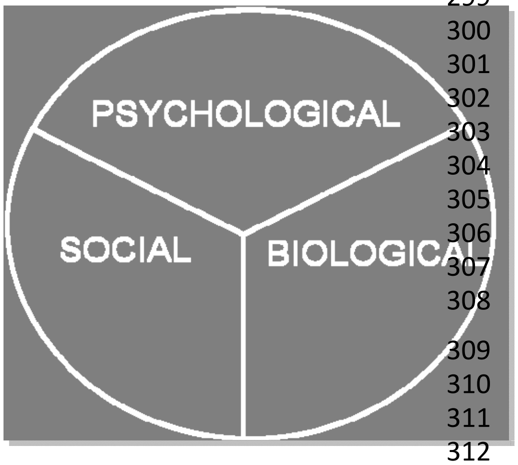

Such management focuses on the interplay among biological, psychological, and social factors that underlie the interventions to be tested and the key outcome domains to be assessed in CAMEO. Applied to CAMEO, the pharmacological (opioid adjustment coupled with co-analgesics) and behavioral (pain self-management/coping) treatments will not simply address the **biological** or physical experience of pain. Rather, CAMEO will be *individually tailored to incorporate patient preferences* all the time taking into account how **psychological** and **social** factors are intertwined to influence pain severity, functional interference, and response to treatment.

Collaborative care between veterans, nurses, and psychologists is fundamental to the integration of biological, psychological, and social factors in both intervention arms. Nurse care managers and clinical psychologists are trained to: 1) pay special attention to patient attitudes, beliefs, and illness behaviors that may influence pain severity, functioning, and adherence to treatment; 2) assess patients for opioid misuse and psychological distress, especially depression and anxiety symptoms, which are frequently co-morbid with chronic pain; 3) monitor for adverse effects from treatments delivered; and 4) create a supportive patient-clinician relationship and social environment that helps veterans cope more effectively with their pain and to take a more active role and shared responsibility in their management. Additionally, pain self management/coping skills address psychological aspects of chronic pain.

Since chronic pain also demands a combination of health system and organizational elements for effective management, the Chronic Care Model[60] also provides some theoretical under-pinning for CAMEO. Of the six Chronic Care Model elements, ***delivery system design*** (care management, primary care-mental health integration)**, *self-management support*** (pain self-management/coping skills)**, *clinical information systems*** (information technology, i.e. CPRS applied at the point of care between care managers and patients), and ***decision support*** (medication algorithms that guide treatment decisions) are relevant to our study. Furthermore, the CAMEO interventions are designed to facilitate “productive interactions” between patients and providers to improve clinical outcomes for patients with CLBP.

D2. OVERALL DESIGN

Our study population will be 300 veterans with chronic low back pain (CLBP) who have persistent moderate to severe pain despite long-term opioid therapy. Participants will be recruited from the five primary care clinics at Roudebush VA Medical Center (RVAMC) and three community based outpatient clinics (Bloomington, Terre Haute, and Martinsville CBOCs). CAMEO will be a *2-arm*, parallel group, randomized comparative effectiveness trial. Eligible patients will be randomized to one of two arms: 1) guideline-concordant opioid

management coupled with algorithm-based co-analgesic treatment **(PHARM)**; or 2) pain self-management/coping skills training **(BEH)**. The intervention period will last for 6-months, after which time patients will be followed for an additional 6 months (total of 12 months). Treatment response will be assessed at 6-months post-randomization (immediate post intervention) and at 9- and 12-months post-randomization (to assess for sustained responses). CAMEO will last 4 years including 6 months for start-up; 2.5 years for recruitment; and 1 year for follow-up, data analysis and manuscript preparation. **Figure 2** shows the CAMEO trial design.

D3. JUSTIFICATION FOR STUDY DESIGN

To justify the CAMEO study design we address the following questions: (1) *why no control group* (2) *why a head to head comparison between pharmacological vs. behavioral treatments*; (3) *why will nurses deliver the PHARM treatment arm and clinical psychologists deliver the BEH treatment arm*; and (4) *why a multi-site trial*.

(1) *Why no control group?* We decided to jettison the usual care control arm from our previous proposal for two reasons. First, the revised application is framed more squarely as a comparative effectiveness study in response to reviewer recommendations. Second, findings from both SCAMP and APT trials have provided convincing evidence that a "usual care" control arm is ineffective for chronic pain management in the primary care setting.

(2) *Why a head to head comparison between pharmacological vs. behavioral treatments?* While there is trial evidence supporting these treatments, the effectiveness of both approaches has not been convincingly demonstrated in the primary care setting. Also, these approaches have not been compared against each other and our current design is a significant and innovative step beyond our previous (SCAMP) and ongoing pain trials (ESCAPE and SCOPE). We believe a head-to-head comparative effectiveness study design best answers the question how to most effectively treat CLBP, especially for veterans with CLBP refractory to opioid therapy. In addition, patients frequently differ in their preferences with respect to pharmacological and behavioral treatments, and a trial to determine the clinical effectiveness and cost-effectiveness of two evidence-based treatment options for CLBP has immense importance. This population of veterans is particularly challenging to PCPs and alternative treatments are critically needed.

(3) *Why will nurses deliver the PHARM treatment arm and clinical psychologists deliver the BEH treatment arm*? Delivery of the interventions by different clinicians will avoid the potential problem of contamination across treatment arms raised by reviewers in our last application. Most importantly, the two treatments represent two different delivery models already embedded in VA primary care: 1) nurse care management; and 2) mental health-primary care integration (co-location of clinical psychologists and PCPs). Both delivery models hold promise to decrease the time needed to deliver algorithm-based analgesics or pain self-management/coping skills and monitor treatment response, side effects, and adherence. Findings from CAMEO will help clinical managers, hospital leaders, and policy makers make operational decisions related to system redesign, care delivery implementation, and resource allocation based on local facility strengths, resources, and patient preferences.

(4) *Why a multi-site trial?* We added three new study sites compared to the last application to improve generalizability, to avoid saturating or "exhausting" the potential study participant pool, and to extend our intervention to CBOCs, an important source of primary care within the VA nationally.

D4. STUDY SITES

The RVAMC is an urban, university-affiliated, tertiary care center which provides health care for more than 53,000 veterans and houses five primary care clinics. *The Indy West Clinic, an RVAMC expansion clinic, opened its doors to patients in January 2010 and will serve as a new study site.* The clinics staff 75 primary care providers (faculty and resident physicians; advance practice nurses), caring for over 20,000 patients who make over 65,000 visits per year. *The RVAMC is also the parent facility to three community based outpatient clinics—the Bloomington, Martinsville, and Terre Haute Outpatients Clinics. The Bloomington and Terre Haute CBOCs staff five primary care providers at each site to provide primary care, prescription and other services to approximately 8,500 veterans in the Bloomington and Terre Haute, Indiana metropolitan areas (approximately 1 hour drive from Indianapolis).* The Martinsville CBOC will open in October 2011 and be initially staffed by one primary care provider. *See support letters from Drs. Kalu and Parmar, the Medical Directors at Bloomington and Terre Haute CBOCs, respectively.*

D5. RECRUITMENT

To plan for recruitment and assess feasibility, we conducted a search of CPRS to determine the number of RVAMC veterans with at least one primary care visit in the past 2 years (using clinic stop codes), who had low back pain (i.e., ICD-9 721.x; 722.x, or 724.x), and were on long-term opioids--defined as 3 or more prescriptions (of ≥ 28 days) in the past year. We identified *2,460* unique veterans who met all "feasibility" criteria. Based on our SCAMP and ESCAPE trials, we have found that > 50% who meet the initial feasibility criteria also meet our study eligibility requirements, which will give us a potential population of over *1,200* patients from which to recruit 300 participants. Our medical center is also expecting more than 8,000 new enrollees from the Iraqi and Afghanistan conflicts alone for fiscal years 2010 and 2011. Thus, there are a sufficient number of potential participants available for this study, especially with the addition of three new recruitment sites. Furthermore, since SCAMP was completed in June 2008 and ESCAPE will finish enrollment in September 2010, these studies will not "compete" for participants with CAMEO.

We have successfully enrolled patients for several moderately sized trials at our medical center. For example, in the SCAMP trial we successfully recruited over 65% of participants contacted in the VA. In our CRAFT study of primary care patients, 100 (84%) of 119 eligible patients who were approached were successfully enrolled. In our "Pain as the 5th Vital Sign" pilot study, 159 (77%) of 206 participants surveyed expressed a willingness to participate in a clinical trial to treat their pain if offered such an opportunity. Based on these prior successes, we anticipate no difficulty meeting our sample size requirements. In terms of retention, we assessed 87% (217/250) of SCAMP participants at 6-months and 82% of participants (205/250) at 12-months.[40]

Consistent with previous studies conducted at RVAMC, the CAMEO study sample will include approximately 5% to 15% women and at least 15% minorities, thus reflecting the demographics of RVAMC. In our SCAMP study, African-Americans and women made up 16% and 6% of the veteran sample, respectively. We do not plan to over-sample women or power sufficiently to explore sex differences in treatment response. The racial/ethnic composition of RVAMC and participating CBOCs makes exploring racial differences in treatment response impractical unless we employed special efforts to recruit minorities other than African-Americans.

D5a. Identifying and Enrolling Potential Participants

PCPs will be informed of CAMEO study details and will be asked to provide signed approval so that our team may contact their potentially eligible patients for participation in the trial. The CAMEO research team, not the PCPs, will determine eligibility by applying the inclusion/exclusion criteria to potential participants during an "eligibility interview." In our previous trials, over 95% of physicians have agreed to allow us to approach their patients. Potential participants will primarily be identified by querying CPRS to create a master list of veterans who meet the following criteria: 1) primary care visit in past 2 years; 2) moderate pain severity (pain score ≥ 5); and 3) long term opioid use (3 or more prescriptions for opioids in previous 12 months).

This list of potential participants will be updated monthly during the enrollment period and a recruitment letter, signed by their PCP, will be mailed to qualifying veterans to describe the study. Potential participants will be contacted by phone within a week after receipt of the letter to assess eligibility and determine their interest in participating. If the veteran is eligible, an appointment will be scheduled to obtain a signed informed consent statement (**Appendix 1**) and HIPAA authorization from those who desire to participate. The baseline interview will be conducted at this time.

This method of identifying potential study subjects through CPRS and contacting them for possible study participation has been approved by both our university IRB and VA Scientific Review Committee for the SCAMP (which enrolled 200 veterans) and ESCAPE trials (which will enroll 240 veterans). A second method of enrollment will involve inviting veterans who are enrolled in our IRB-approved Pain Registry, which includes subjects from our previous pain studies, to participate in the study. A third method, if needed, will be in-clinic contact of potential subjects by cross-referencing the CPRS list with the weekly appointment roster for each participating PCP. A fourth method is self-referral by patients responding to study advertisement displayed in the primary care and post-deployment health clinics and hospital elevators.

D5b. Eligibility

Veterans will be eligible if they have: 1) CLBP of at least moderate intensity; 2) pain for ≥ 6 months; 3) on chronic opioid therapy; and 4) access to a working telephone. Exclusion criteria includes: 1) severe medical conditions; 2) active psychosis; 3) schizophrenia; 4) active suicidal ideation; 5) pending back surgery; 6) moderately severe cognitive impairment; 7) involvement in ongoing pain trials; and 8) pregnant or trying to

become pregnant. We will exclude veterans with an active substance use disorder (i.e., those currently in treatment), but to maximize generalizability we will not exclude those with a past history of substance abuse. These latter patients are at higher risk for opioid misuse and abuse and thus require more intensive monitoring during the study. To further maximize generalizability and expand our potential sample size, we decided to not exclude veterans with current (or applying for) disability (service-connected or social security) for CLBP.

Since the SCAMP trial was completed in June 2008 and ESCAPE will be completed this fall, veterans who participated will be potentially eligible for CAMEO. However, current participants in the SCOPE trial will not. Access to a telephone (landline or cell) is required because most of the outcome assessments will be conducted via phone. Exclusion criteria will be determined during the baseline eligibility survey *conducted by our study team (not the PCP)* and are designed to eliminate potential participants for whom the proposed interventions are inappropriate or unsafe and/or for whom there may be disincentives for improvement. These include severe medical conditions that may limit participation: (1) significant cardiovascular disease: NYHA functional class 3 or 4 congestive heart failure; systolic blood pressure ≥ 180 or diastolic blood pressure ≥ 105 mmHg; myocardial infarction, stroke, or TIA within 6 months; chest pain or dizziness with exercise; (2) COPD or asthma needing home oxygen; (3) cancer (other than skin cancer) receiving treatment or treatment planned in the next 6 months. Moderately severe cognitive impairment defined by a 6-item validated screener.[61]

D6. RANDOMIZATION

After providing written-informed consent and completing their baseline interview, participants will be randomized to one of two arms*:* 1) guideline-concordant opioid management coupled with co-analgesic medications (PHARM)*;* or 2) pain self-management/coping skills training (BEH). Randomization, at the patient-level, will be done by Christy Sargent, BS (project coordinator) using sealed, opaque envelopes to maintain allocation concealment. Group assignment will be determined from a table of random numbers created by Xinli Li, PhD, MS (statistician) in blocks of 3 and 6.

D7. CAMEO INTERVENTION DETAILS

The CAMEO interventions will last 6-months. This duration is predicated on the likelihood that prospective adjustment of medications will be required to optimize pharmacological treatment and pain self-management/ coping skills will require time for the patient to learn and apply to optimize behavioral treatment for CLBP. The length of the follow-up and schedule of outcome assessments at 3, 6, 9, and 12 months are to detect three types of treatment effects: 1) “early” (3-months) intervention benefit; 2) immediate post-intervention benefits at 6-months; and 3) sustained benefits at 9- and 12-months post-randomization.

Medications are the most common mode of treatment for chronic low back pain in primary care. However, monitoring of treatment response with appropriate adjustments (e.g., maximizing doses or switching medications), and assessing adherence, side effects, and signs of misuse (i.e., aberrant behaviors) is often suboptimal in clinical practice. Many patients continue to have inadequate pain relief and poor functioning despite long-term opioids. PCPs need other treatment options if their patients’ CLBP does not respond to opioids or if intolerable side effects emerge. Guided by the biopsychosocial model, effective pain management should encompass more than pharmacological management directed at pain scores; it should address a variety of psychological, social, and behavioral factors that contribute to their pain experience. Nurse care managers or clinical psychologists, working in concert with PCPs, may be in an ideal position to identify these factors and deliver interventions that relieve veterans’ pain. Because of time constraints routinely faced by PCPs, and the complexity involved to effectively manage chronic pain, system support for PCPs, such as nurse care management and mental health-primary care integration (co-location of clinical psychologist), are critically needed in the primary care setting. Nurse care management for optimized pharmacological management and psychologist-delivered optimized behavioral/psychological treatment are central to CAMEO.

D7a. <u>Nurse Care Management for Patients in the Pharmacological (PHARM) Treatment Arm</u>

Generic aspects of nurse care management have proven effective in multiple trials, including monitoring symptoms, assessing adherence, addressing adverse effects, communicating with the primary care physician, and staffing cases with supervising physicians. The PHARM treatment arm is designed to optimize pharmacological treatment for CLBP in the primary care setting. Nurse care managers (NCMs) will deliver guideline-concordant opioid management coupled with algorithm-based co-analgesic treatment (PHARM). We have successfully trained NCMs to provide pharmacological management (antidepressant therapy in two trials (AIM—for post-stroke depression and SCAMP) and analgesic therapy in one trial (ESCAPE).

The NCMs will meet weekly with physician and pharmacist-investigators (Drs. Bair, Krebs, and Zillich) to review cases and provide advice on treatment plans. We have successfully implemented this model of case supervision in four other clinical trials. Also, a physician-investigator (usually Dr. Bair) will be available at all times to discuss any management issues that arise between the weekly case meetings.

D7b. Schedule of Nurse Care Manager Contacts with Patients in the Pharmacological Treatment Arm

The timeline of NCM contacts with patients in the PHARM arm is outlined in **Appendix 2**. Patients will receive at least 9 contacts with the NCMs over the trial period. Participants will have an initial visit at baseline to assess their current and past treatments for CLBP, pain intensity, and pain-related limitations. Patients' opioids will be adjusted and/or co-analgesics will be initiated. During follow-up calls, patients' pain severity, response to treatment, adherence, adverse effects, and desire to change current treatment will be assessed. Follow-up NCM telephone contacts will occur at 2, 4 and 6 weeks after baseline, and months 2, 3, 4, 6, and 9 months. On average, these calls last between 10 to 20 minutes. In a focus group study, SCAMP participants found these calls of high value and did not perceive them as burdensome.[47]

Making the intervention reproducible requires an algorithmic approach. For this reason, a minimum of 7 contacts will follow the initial assessment. From previous studies of care management interventions, however, we know that flexibility is critical to helping some patients make treatment changes and improve clinical outcomes. We therefore leave some discretion to the NCMs as to whether and when to make supplemental patient contacts. Also patients sometimes initiate contact with them. Detailed logs will be kept of the timing and content of patient contacts so that we can describe their activities and measure the "intervention dose" in reports and papers from CAMEO.

D7c. Guideline-Concordant Opioid Management for Patients for Patients in the Pharmacological Arm

In clinical practice "doing well" on long-term opioid therapy means: (1) achieving meaningful pain relief; (2) improving one's ability to function; (3) experiencing minimal or no side effects on steady doses; and (4) adhering to the rules of opioid therapy outlined in an opioid treatment agreement.[49] Successful chronic pain management involves balancing the appropriate use of opioids with the prevention of misuse and abuse. While abuse potential is real, and stories of prescription opioid abuse are frightening to PCPs, addiction during long-term opioid use is relatively rare. For some patients these medications do offer benefits when they are properly prescribed and used in accordance with VA/DoD opioid guidelines.[49] Additional principles of opioid treatment, which have not been a focus of our other trials, will be employed in CAMEO, and include:

1. Participants will be given a reasonable "opioid trial," i.e., continuing an opioid or trying two or more agents in order to find the best balance between relief and adverse effects.
2. Long-acting opioids will be initiated at low doses in patients who have not responded to or experienced only partial effectiveness from short-acting agents.
3. Long-acting morphine will be used as the first-line long-acting opioid.
4. Methadone will be considered if morphine is ineffective or leads to bothersome side effects.
5. Long-acting opioids will be titrated in a conservative and measured way (until a stable dose is reached) at interval visits if only partially effective.
6. Short-acting opioids will be considered for breakthrough pain.
7. Patients not doing well on one opioid may be tried on another (opioid rotation).

Multiple guidelines recommend that patients on long-term opioids need to be monitored regularly, documenting changes in pain intensity, functioning, and behaviors that may predict misuse and abuse.

D7d. Opioid Adherence Monitoring

The VA/DoD Clinical Practice Guideline for the Management of Opioid Therapy for Chronic Pain includes detailed recommendations for opioid monitoring.[49] The overall goal of monitoring is to ensure effective and safe use of opioids. The risks of opioid misuse and abuse are not well understood. "Misuse" is defined as the use of opioids in a manner other than indicated or prescribed. Abuse occurs when use is detrimental to the individual or others, or when opioids are used unlawfully. "Diversion"—the selling, sharing, or trading of prescription opioids--is an example of abuse. Taking steps to minimize the risk of misuse and abuse is prudent and must be accomplished by appropriate screening and risk management tools. Others recommend a "universal precautions" approach for all patients on opioid therapy,[62] because, by themselves, provider "intuition" and patient self-reports are inadequate to accurately detect "at risk" patients for opioid abuse.[62]

Although the efficacy of these tools remains unproven, their inclusion in guidelines and clinical policy is widespread. Despite this inclusion, these tools are not routinely used. In a chart review study conducted by Dr. Krebs, these tools were used only 15% of the time at our VA. The most commonly used tools include obtaining informed consent for chronic opioid therapy, using opioid contracts or agreements, and performing random urine drug tests. We will assess the risk of misuse, including possible diversion, in CAMEO participants through review of their clinical history and medications, validated questionnaires, and urine drug tests will be done randomly at least twice during the study. For the purposes of this study, hair and nail analysis were not considered feasible or practical since these tools are used rarely clinically for toxicology screening. Patients will be asked to sign an opioid treatment agreement (**Appendix 3**) at enrollment. Any opioid related problems observed during the study will be discussed with the patient's PCP to develop consensus on a resolution.

D7e. Protocol to Adjust Opioids or Initiate Co-analgesic Medications during Pharmacological Management

During the baseline assessment, the NCMs will determine current and past treatments for CLBP and establish whether or not patients have had an adequate trial (i.e., were analgesics sufficiently dosed). If not, the NCM (in consultation with Drs. Bair, Krebs, and Zillich) will recommend an adjustment of the patients' opioid or initiate treatment with a co-analgesic with appropriate dosing and scheduling. Co-analgesics (antidepressants, anticonvulsants, local anesthetics, and other medications) are a diverse group of drugs that have been studied in numerous clinical trials, may enhance the effects of opioids, and have independent pain-relieving properties.

After prescriptions are written or entered electronically by the study physicians, all study medications will be dispensed through the RVAMC pharmacy. The NCM will interact regularly with Mr. Win Turner, RPh, the RVAMC Research Pharmacist, who will oversee study medication dispensing. Participants' PCPs will be integrated as a "partner" and informed of medication changes in two ways. First, when opioid changes are recommended, the NCM or Dr. Bair will page the PCP and speak with them directly about the change. To avoid patient care disruptions these exchanges will be conducted prior or after the PCP's clinic. Second, for co-analgesic changes a study physician will enter a note in CPRS reflecting the change and then "view alert" the PCP to keep them informed. Based on our previous trial experience, we expect disagreements between our research teams' recommendations for a particular opioid or co-analgesic to occur infrequently. However if disagreements arise, consensus will be reached through telephone or face-to-face discussion with the PCP. If by chance consensus is not reached, the PCP's decision will take priority. Analgesics initiated by the PCP will be recorded and tracked by the NCMs and research assistant during the scheduled outcome assessments.

Two weeks after adjustment/initiation of analgesics, the NCM will contact intervention participants by telephone in order to assess response, adherence, and potential side effects. If bothersome side effects have prompted non-adherence, discontinuation, or reluctance on the part of the patient to continue the analgesic(s), the analgesic will be changed. Subsequently, nurses will again assess clinical response at four and six weeks (after baseline) and in months 2, 3, 4, 6, and then at 9 months. Drs. Bair, Krebs, and Zillich will supervise the weekly care management meetings to discuss patients as well as consultation between meetings as needed. **Treatment response** will be evaluated in three domains: (1) pain intensity; (2) pain-related disability; and (3) global improvement. To simulate clinical practice and enhance patient-centeredness of CAMEO, treatment preferences (i.e., desire to change treatment) will also be assessed and considered prior to treatment changes.

The NCM will follow an evidence-based medication algorithm that lists simple analgesics and co-analgesics to guide treatment decisions. We developed this algorithm **(Table 2)** based upon our synthesis of relevant research detailed in a manuscript and two book chapters we published.[56;63;64] While we provide a rational sequence of analgesic selection, we are not testing any particular medication in CAMEO but, instead, are testing the optimal analgesic management that is both effective and tolerated in an individual patient. Since some veterans may require a change in analgesics during the trial, this pragmatic, patient-tailored approach is more similar to clinical practice and approximates the optimal strategy for real-world pain management rather than an inflexible testing of a single drug. If this approach is found to be effective, it could be implemented as a novel delivery model for CLBP since nurse care management is already utilized for other chronic conditions in VA primary care. This structured approach to pharmacological treatment is similar to that effectively applied in our completed SCAMP trial for optimized antidepressant therapy.

D7f. Algorithm-based Co-analgesic Treatment for Patients in the Pharmacological Arm

Based on our evidence-based medication algorithm **(Table 2)**, we plan on maximizing non-opioid analgesics (NSAIDs and acetaminophen) and other co-analgesics before escalating or rotating opioids. Co-analgesics may enhance the effects of opioids and have analgesic effects for CLBP.[56]

Table 2 Step-wise co-analgesic algorithm*

Step 1 Medications:

Simple analgesics

1. Acetaminophen 650mg mg q 6h (max 2000 mg if cirrhosis or ≥ 3 alcoholic drinks/day)
2. NSAIDs: try at least two (except in patients with renal impairment; peptic ulcer disease)
   a. 1st line: naproxen 500 mg q 12h or 500 q am plus 250 bid (max 1000)
   b. 2nd line: (i) salsalate 1000 mg q 8h or 1500 q 12h (max 3000); (ii) etodolac 300 mg q 8h or 500 q 12 h (max 1000); (iii) ibuprofen 600 mg q 6h (max 2400); (iv) piroxicam 10mg qd

Step 2 Medications:

Tramadol

1. Start 25 mg BID or TID and titrate to 100 mg QID (max 300 mg if age > 75; max 100 mg BID if CrCl < 30, 50 mg BID if CrCl <10; max 50 mg BID if cirrhosis)
2. Use concurrent acetaminophen, 500-1000 mg dosed with tramadol TID-QID

Step 3 Medications:

1. Gabapentin, titrate up to 900-1200mg tid
2. Cyclobenzaprine 5-10mg qhs-tid
3. Venlafaxine, titrate up to 225mg qd
4. Duloxetine (60 mg qd) and/or Pregabalin (300-450 mg qd, divided into bid doses)

Step 4 Medications:

TCAs: try at least two

1. Amitriptyline, start at 10-25, titrate to 100 mg (max 50 mg if taking an SSRI/SNRI)
2. Nortriptyline, start at 10-25, titrate to 100 mg (max 50 mg if taking an SSRI/SNRI)

*Avoid amitriptyline in older adults—65+ years: Other safety concerns addressed in **Human Subjects**

These medications include antidepressants, anticonvulsants, topical anesthetics, and muscle relaxants. Since no specific analgesic or medication class has proved superior to another, the pain medication algorithm is a step-wise series of brief individual drug trials, and includes tailored steps depending on the patient's clinical response and preferences. The concept of "rational polypharmacy"[65] will be applied to find the best balance of pain relief and side effects. This concept is based on experience that patients often have improved pain with fewer adverse effects with a combination of moderate doses of medications that work through different mechanisms, instead of high doses of a single agent.[65] We address safety concerns of using these medications in the **Human Subjects** section.

As mentioned above, **treatment response** for patients will be evaluated by the NCMs using scripted questions **(See Appendix 6: Telephone Script for Nurse Care Managers)** in three domains: (1) pain intensity; (2) pain-related disability; and (3) global improvement ("overall, since starting the study, would you say your pain is?") Patient treatment preferences will be frequently considered. If the patient does not improve or improves in only one domain, we will recommend a change in treatment if a change is desired by the patient. If 2 or 3 domains improve we will continue the current plan. For patients requesting regular dose escalations without improvement, we will conclude that they have opioid-unresponsive pain and will probably not benefit from opioid therapy. An appropriate "exit strategy" to discontinue the opioids will be implemented if patients are not achieving appropriate goals of treatment or if they display behaviors (e.g., drug hoarding, aggressive complaining about pain medications, frequently losing prescriptions, resisting changes to medication, despite adverse side effects, unsanctioned dose escalations) that may represent misuse or abuse. Of note, all modifications and titration of patients' opioids will be done with the PCP's approval. For patients whose pain has not adequately responded at the end of the trial, Dr. Bair will recommend that their PCP consider a pain clinic referral or other appropriate specialty consultation (presuming analgesic adherence is confirmed), since a more complex treatment plan may be warranted. At study's end, PCPs will be informed of their patient's medication regimen and subsequent treatment decisions will be left to the PCP's discretion.

D7g. Justification for the Behavioral (BEH) Treatment Arm

Non-pharmacological interventions for chronic pain are generally designed to promote self-management by providing education, recommendations, and support to increase coping skills. Pain self-management interventions foster skill acquisition and practice in an attempt to address patients' attitudes and beliefs, reduce psychological distress, and modify illness behaviors to improve health. The success of self-management interventions is theoretically linked to intermediate outcomes such as self-efficacy. Self-efficacy is defined as the confidence to complete a behavior in order to reach a desired goal and is a strong mediator of behavioral change.[66] When an individual feels confident in performing specific behaviors, the efforts toward achieving the end result are marked by more persistence, higher goal setting, greater problem solving, and more expended effort. Conversely, lacking self-efficacy to manage, cope, or function with pain has been shown to be a strong predictor of pain intensity, disability, and depression.[66] Moreover, self-efficacy beliefs have an independent effect on pain avoidance behaviors and disability even after controlling for pain severity and depression.[67]

Patients with chronic pain often develop maladaptive cognitions/thoughts (i.e. catastrophizing) and behaviors (i.e., fear of movement) that contribute to physical and emotional suffering. To manage these challenges, cognitive-behavioral interventions teach patients' ways to identify and change maladaptive thoughts, feelings, and behaviors and to replace them with those that are more helpful to cope with their pain. In the context of CAMEO, clinical psychologists will help participants develop pain self-management skills and challenge maladaptive cognitions and modify dysfunctional beliefs veterans may hold regarding their pain-related disabilities and interpretations of their pain experience. Participants will learn and maintain more adaptive ways of thinking about themselves and their CLBP to reduce pain and disability.

In Morley's review[20] of randomized controlled trials of cognitive-behavioral interventions for chronic pain, the median number of treatment hours was 16 (range 10-18/h). In recent years, the field of psychology has moved towards eight or fewer therapy sessions to address the barriers of resource constraints, insurance coverage challenges, and the fact that most psychotherapy clients attend less than eight sessions.[68] Turner et al. demonstrated the benefit of brief cognitive-behavioral therapy (CBT) in temporomandibular pain,[69] Similarly, brief CBT (i.e. 6-8 sessions) improved physical functioning in patients with fibromyalgia.[70] Another recent advance has been the advent of telephone based interventions for arthritis[71] and depression care.[72] Telephone based interventions have the advantage of covering multiple, geographically-dispersed veterans and practices.

D7h. <u>Overview of Pain Self-Management/Coping Skills Training in the Behavioral Treatment Arm</u>

Table 3. Pain Self-Management/Coping Skills

- Overview and causes of CLBP
- Identifying pain triggers and influences
- Handling pain flare-ups
- Increasing physical activity
- Goal Setting and planning
- Problem solving
- Overcome fear of movement/re-injury
- Positive thinking
- Activity-rest cycling
- Scheduling pleasant activities
- Relaxation and deep breathing
- Attention-diversion techniques
- Tips for better sleep
- Effective communication with providers
- Reframing or changing cognitions

Veterans randomized to behavioral treatment arm will receive a series of approximately 9 pain self-management/coping skills training sessions delivered by one of three primary-care based clinical psychologists. The BEH intervention, especially the pain self-management skills manual **(Appendix 4),** was developed partly from our SCAMP trial and proven effective in Dr. Damush's primary care trial of low back pain[22] as well as arthritis trials by Lorig, and Von Korff.[73;74] To compliment the behavioral focus of self-management, the pain coping skills training will draw upon a manualized cognitive-behavioral program **(Appendix 5)** applied in our ESCAPE trial, modeled after previous CBT interventions,[75] and empirically validated in prior studies of pain. Self-management training will focus on increasing self-efficacy to manage low back pain and *coping skills training will focus on the basic CBT concept that pain is a complex experience affected by thoughts, feelings and behaviors.* Since optimal application of non-pharmacological interventions for pain involves tailoring to patient needs,[76] participants will be introduced to a "menu" of self-management and coping skills **(Table 3)** rather than receive a prescribed program. Delivery of the behavioral intervention will employ a flexible approach that is easily adapted to individual preferences and perceived need for learning specific pain coping skills. Tailoring will include the selection of relevant content and skills and assessment of readiness to change behaviors.[77] Patients will choose skills to learn and behaviors to modify that they perceive most relevant to them.

Participants will learn to modify and sustain healthy behaviors through goal setting and problem-solving techniques. Barriers to engaging in self-management behaviors will be discussed. Each session will involve a

discussion of patients' thoughts and feelings about their pain, past treatments for pain, and identification of barriers to reducing pain severity and interference with activities. To facilitate these discussions, we will draw upon two models. The first is Emery's "4 A's model" to help veterans modify dysfunctional cognitions related to pain.[78] Participants are asked to be **aware** of dysfunctional cognitions; **answer** dysfunctional cognitions (restructure); **act** on the more accurate and/or helpful beliefs; and **accept** imperfection. The second is a problem solving model to frame and overcome perceived barriers to inadequate pain improvement. This model has five steps: 1) identify the problem; 2) brainstorm to think of possible solutions; 3) rank solutions from most to least promising; 4) implement the solution ranked #1; and 5) evaluate—"Did it work? If not, why not?

D7i. <u>Structure and Content of the Behavioral Treatment Arm Sessions</u>

The sessions will last a maximum of 45-minutes to optimize participants' attentiveness and performance required by the cognitive demands of pain coping skills training. Each session will adhere to a common structure organized into three parts: 1) check-in; 2) intervention, and 3) wrap-up. Prior to each session participants will be asked to rate the strength and perceived impact for up to four pain beliefs that participants and the psychologist identified together **(See Appendix 7: Telephone Script for Clinical Psychologists).** . This exercise sets the stage for problem identification and provides a bridge from the last session. The check-in includes welcoming, a brief pain update on progress and concerns, and collaborative agenda setting of at least one priority item to provide structure for the session. The intervention represents the bulk of the session and includes a discussion of old and new barriers identified while applying self-management and pain coping skills. For example, this generally includes addressing a participant's select dysfunctional cognitions about pain and its impact by disputing their accuracy, and developing a more adaptive cognition (i.e. cognitive restructuring). The wrap-up involves patient refection on what was and was not helpful, a summary, collaborative goal setting for the next session, and evaluate progress through ratings of select cognitions and practice assignments. The purpose of these assignments is to apply lessons learned and help assess understanding of the material. To track the success of interventions and provide a focal point of discussion during sessions, participants will rate the accuracy of the dysfunctional and alternative cognition which are tracked on two self-monitoring forms. Patients will receive individualized feedback from the psychologists about their progress.

The pain self-management/coping skills training sessions will be delivered by psychologists using standardized, written manuals included in **Appendix 4 and 5**. The sessions will occur during the scheduled clinical contacts (by telephone or face-to-face depending on patient preferences) at within one week of the baseline, 2 4, and 6 weeks, and months 2, 3, and 4 and skills reinforced at months 6 and 9. The content of these sessions are designed to modify coping strategies found to be related to pain and disability. Briefly, patients will be trained in a variety of evidence-based skills found to help reduce pain and improve function. For example, patients will be trained in three attention diversion methods: relaxation, imagery, and distraction. Relaxation training, using a protocol and relaxation tape described by Surwit,[79] will involve concentrating on muscle tension signals and using them as cues to relax and has been successful in managing negative mood. Imagery will be taught as an adjunct to relaxation.[80] Patients will practice using pleasant imagery and changing from one image to another. Distraction techniques will include focusing on physical or auditory stimuli.[80] Another skill that will be introduced to participants is activity-rest cycling and pleasant activity scheduling [81-83] which enables patients to pace and increase their activity level. In activity-rest cycling, patients identify activities in which they overexert themselves (e.g., yard work, home repairs), learn to break those up into periods of activity and rest (e.g., 45 minutes of yard work followed by 10 minutes of rest), and gradually increase their activity level as they decrease rest. Patients will identify activities they enjoy such as reading, doing hobbies, or visiting friends and set and record weekly activity goals. Each patient will develop a written maintenance plan that includes a list of coping skills, home practice, and a plan for dealing with setbacks and pain flare-ups.

D7j. <u>*Steps to assess and assure treatment fidelity in the behavioral arm*</u>

We will take a number of steps to ensure that the treatment protocol for pain self-management/coping skills training is delivered uniformly by all treatment providers involved in the study. First, all pain self-management/coping skills training sessions will be implemented by clinical psychologists with experience treating patients with chronic pain. Second, all psychologists who participate in the study will receive pain self-management/coping skills training through workshops facilitated by Drs. Damush and Bair. Third, all psychologists will be provided with detailed treatment scripts, and the treatment strategies will be taught through didactic instruction, taped illustrations of techniques from model cases, and role-play of common

scenarios. Fourth, the psychologists will document treatment delivery details (content, time, mode of delivery). Finally to provide supervision, 20% of sessions will be audio-taped and investigators will review tapes for representative initial, middle and ending sessions for each psychologist during the study to assure that procedures are followed. Remedial training will be provided if needed i.e. deviation from protocol.

D8. DATA COLLECTION PROTOCOL

The schedule of a comprehensive set of outcomes and key variables to evaluate the effectiveness of the CAMEO interventions are listed in **Table 4**. After obtaining informed consent, a research assistant will administer an in-depth baseline assessment to gather socio-demographic data, review the patient's history with an emphasis on previous treatments tried for their pain, and administer several validated measures of pain, disability and psychological status. All patients will undergo a targeted physical examination, including the objective functional measurements of strength, range of motion, and flexibility, by one of the study personnel. The data collection protocol is informed by Initiative on Methods, Measurement and Pain Assessment in Clinical Trials (IMMPACT) recommendations,[84] biopsychosocial conceptual model, and our previous studies.

The baseline interview will take approximately 45 minutes, the 3, 9, and 12-month interviews about *20 minutes*, and the 6-month *(primary outcome time point)* interviews about *30 minutes*. These assessments will be completed by our research assistant and conducted by telephone, except for the baseline and 6-month interviews, which are done in person to establish rapport with participants and to perform the functional measures. Additionally, we have found phone interviews over 30 minutes to be burdensome to both patients and interviewers. We have used a battery of measures of similar length and administration time in several previous or current trials without over-burdening patients. All measures have been conducted both in person and by phone in multiple prior trials. Both types of administration have routinely been approved by both Indiana University IRB and VA Scientific Review Committee. The baseline and follow-up outcome assessments will be conducted by research assistants blinded to treatment assignment.

If participants cannot be reached by phone we have employed two strategies to capture all outcome assessments: (1) send a mailed questionnaire to the veteran with postage paid, self-addressed envelope to our office; and 2) conduct a face-to-face interview in conjunction with the patient's clinic visit. Veterans occasionally lack transportation to the in-person interviews. In this situation, we have arranged taxi cab rides to and from our VA. To protect against data loss, participant responses are collected in two formats: paper and electronic. The interviews and study databases will be designed in Microsoft Access by Mr. Larry Yang, MS, VA data manager. To maintain confidentiality of veterans interviewed, our research assistants will adhere to careful interview and data collection procedures. First, participants will be told that their responses will remain confidential and that every effort will be made to fulfill that assurance. Second, the interviews will be conducted in an appropriate setting (i.e., private interview room). Third, completed surveys will be stored in a secure location in our Center in a locked file cabinet.

D8a. Primary Outcome Measure

The Brief Pain Inventory (BPI) was developed to assess the severity of pain and the impact of pain on daily functioning, and has been validated in primary care studies.[40;85] The BPI is an 11-item measure that provides scores for pain intensity and pain-related functional impairment. The **BPI pain intensity score** is an average of four ratings of 0 ("no pain") to 10 ("pain as bad as you can imagine") for current, least, worst, and average pain in the past week. The **BPI pain interference score** averages seven ratings 0 ("does not interfere") to 10 ("interferes completely") of interference with general activity, mood, walking ability, normal work, relations with other people, sleep, and enjoyment of life. The **BPI total score** is the average of the intensity and interference scores to provide a summary score. The BPI will be used as the primary outcome measure for several reasons. First, it has been shown to have strong internal consistency both in its original validation study (Cronbach's alpha = 0.77) as well as in our SCAMP study (alpha = 0.83 for BPI severity and alpha = 0.88 for BPI interference).[40;85] Second, it has proven to be sensitive to change in our SCAMP trial (absolute difference in BPI total score between intervention and control groups of 1.39, p = <.001, effect size of 0.49).[40] Third, the BPI assesses the two most important domains (severity and interference) recommended for pain studies.[84]

In addition to our main outcome measure of pain severity and pain interference with activities, we will also measure several other secondary outcomes recommended by the IMMPACT guidelines,[84] and consonant with the biopsychosocial model at each follow-up assessment. These include depression, anxiety, health related quality of life, pain beliefs, *social support*, and substance misuse. These variables are important to assess as

key moderators or mediators of intervention effects. We will also assess opioid-related adverse effects with the rationale that optimized pharmacological or behavioral treatment may limit these side effects. Since the incidence and prevalence of opioid misuse in patients treated for chronic pain is unclear and remains a topic of debate, this will be examined with complementary methods (self-report and urine drug testing). Finally, objective functional measures assessing back strength, flexibility, and range of motion will be assessed.

D8b. Measures, Schedule, and Mode of Administration

Baseline patient characteristics will be evaluated with an interview adapted from our previous trials and will include socio-demographics, disability compensation, comorbid medical and psychiatric disorders, and prior treatments for pain. We will also regularly assess use of other treatments for chronic pain (other medications, exercise, physical therapy, complementary and alternative medicine modalities, and interventional modalities) as well as prescription medication use. For medications, we will record prescribed doses, administration schedule, and number of pills prescribed. We will also assess concurrent prescriptions, especially antidepressants and benzodiazepines.

D8c. Description of Specific Measures

Pain Severity and interference with activities will be assessed with the Brief Pain Inventory (BPI).

Pain-related disability: The Roland Disability Scale is a 24-item pain-specific measure of physical disability originally validated in patients with back pain.[86] It has been used widely in low back pain trials because of its high degree of reliability, validity, and responsiveness to change.

Psychological symptoms are frequently associated with chronic low back pain and will be assessed by:

The PHQ-9 will be used to assess depression severity. Several studies have validated the PHQ-9 as a diagnostic measure with excellent psychometric properties.[87] Internal consistency has consistently been shown to be high (Cronbach's alpha > 0.80) and test-retest assessment showed the PHQ-9 to be a responsive and reliable measure of depression treatment outcomes.

The GAD-7 has demonstrated reliability (alpha = 0.89) and validity (criterion, construct, factorial, and procedural) as a measure of anxiety in the general population and primary care.[88]

The Primary Care PTSD Screen (PC-PTSD) has been validated for use in primary care. The sum of the 4 yes/no items yields a score ranging from 0 to 4, with scores ≥ 3 considered positive for active PTSD. Sensitivity is 78% and specificity is 87% compared to clinician interview.[89]

The PCL-17 is derived from the DSM III-R criteria for PTSD, and is used for diagnosis and as a severity measure. The PCL has demonstrated sensitivity and specificity >70%.The AUDIT-C has been validated as an effective screening test and diagnostic tool for alcohol misuse in primary care sample. A review concluded that the psychometric properties of the AUDIT, such as test-retest reliability and internal consistency are favorable.[90]

Opioid Misuse: The COMM (Current Opioid Misuse Measure) is a 17-item instrument designed to monitor misuse and aberrant behaviors in patients prescribed opioids. It has excellent internal consistency and test-retest reliability. ROC curve analysis showed good accuracy for detection of opioid misuse.[91]

Urine drug screens will be employed *at random* during the study for two reasons: (1) to detect substances that should not be present in the urine; and (2) to detect the absence of prescribed opioids.

Opioid Side Effects: The Numerical Opioid Side Effect (NOSE) is a new 10-item tool designed to assess the most common opioid-related side effects (e.g., nausea, constipation, sleepiness).[92]

Other secondary outcome measures and potential predictors of treatment response will be assessed**:**

The Patient Health Questionnaire stressor scale is a 10-item scale of common stressors scored 0 to 20 which has been used in large primary care studies.

The Medical Outcomes Study SF-36 is well-established generic measure of health status[93] that assesses physical and mental functioning in 8 domains and gives highly reliable, valid and responsive summary scores.

The Pain Catastrophizing Scale a 13-item scale that assesses catastrophizing—a pain belief that have been found to be strong predictor of poor treatment response.[94] Validation studies examining the PCS have found strong evidence of criterion-related, concurrent, and discriminant validity.

The Centrality of Pain Scale is a10-item measure to measure how central chronic pain is to a patient's life.

Pain self-management behaviors will be assessed with 7 items that proved most useful in the pain self-management program in the SCAMP trial in terms of patient uptake and association with treatment outcomes.

Selected items from The PROMIS-28 profile will be administered for pain, fatigue and sleep.

The McGill Pain Questionnaire is a 15-item measure for describing and measuring pain in subjects.

Substance use problems will be assessed by asking questions about the use of others' prescription drugs or street drugs.

The Patient Global Impression of Change (PGIC) is a single item measure to assess overall clinical response. This well-validated and reliable global rating scale is often used as the gold standard for determining clinically important differences for pain outcomes.[95]

Opioid dose—we will assess mean daily opioid dose in milligram equivalents of morphine at baseline, 6 and 12-months to compare the percent of patients in each study arm who had their opioid dose decreased/ increased during the course of the study.

Health Care Utilization and Costs: will be assessed at the end of the 12-month study period for each patient using complete data available in CPRS. This will include all outpatient and emergency room visits, inpatient days, radiographic and lab tests, medical and surgical consultations, drugs, and procedures for low back pain.

Functional Improvement Measure: Gottlieb et al[96] developed a functional improvement measure for patients with low back pain who were undergoing rehabilitation. This instrument is a performance-based measure that can quantify functional capacity and can help identify the level of change associated with treatment.

Table 4. Outcome Assessment Protocol: Measures and Schedule of Administration

**Domain**	**No**	**Measure***	**Items**	**Time (min)**	**Schedule**				
					**BL**	**3 mo**	**6 mo**	**9 mo**	**12 mo**
**Covariates**	1	Demographics; contact information; disability compensation, comorbidity; pain treatment	36	10	X				
**Pain severity/ interference**	2	Brief Pain Inventory	11	3	X	X	X	X	X
**Pain disability**	3	Roland Disability Scale	24	6	X	X	X	X	X
**Psychological**	4	PHQ-9 Depression	9	2	X	X	X	X	X
	5	GAD-7 Anxiety	7	2	X	X	X	X	X
	6	VA PTSD Screener	4	1	X		X		
	7	VA PTSD Checklist (PCL-17)	17	4	X		X		
	8	AUDIT-C	10	3	X		X		X
**Stress**	9	PHQ Stressor Scale (PSS)	9	3	X		X		X
**Opioid Misuse**	10	Current Opioid Misuse Measure (COMM)	16	4	X		X		X
	11	Urine Drug Screen	NA	NA					

**Side effects**	12	Numerical Opioid Side Effect	10	2	X	X	X	X	X
**Generic HRQL**	13	SF-36	3613	3	X		X		X
**Pain beliefs**	14	Pain Catastrophizing Scale	10	3	X		X		X
**Pain coping**	15	Centrality of Pain Scale	10	3	X		X		X
**Response**	16	Global Rating of Change	1	1		X	X	X	X
**Opioid dose**	17	Morphine mg equivalents	NA	NA	X		X		X
**Back Function**	18	Functional Improvement Scale	NA	10	X		X		
**Pain self-mgt behaviors**	19	SCAMP Self-Management Behaviors (SMB)	7	2	X		X		X
**Health Related quality of life**	20	PROMIS profiles – pain (12), fatigue (4), sleep (4)	20	5	X		X		X
**Pain intensity**	21	McGill Pain Questionnaire	15	3					
**Substance use**	22	Illicit use	6	1	X				

D8d. <u>Other treatments: Co-interventions</u>

Participants will continue to be followed by their PCP for all medical care not related to the trial including continuation of other medications as prescribed, clinic visits, and other care. Specifically, use of analgesic medications (both over the counter and prescribed) will be permitted (and assessed), both to adjust for co-intervention differences between groups in the analyses and to assess as secondary outcomes. For example, decreased opioid requirements in both treatment arms may be one indicator of better pain control. All prescriptions, clinic visits, diagnostic tests, referrals, and hospitalizations will be collected using CPRS VISTA.

D9. STATISTICAL CONSIDERATIONS

CAMEO will involve a 2-arm, parallel-group, randomized trial design with two active treatment arms being compared. Since the patients of this trial are not blinded with respect to treatment assignment, it is possible that the observed outcome (if positive) is partially attributed to expectation bias. We will randomize at the patient rather than the physician level for two reasons: (1) randomization at patient level saves sample size; and (2) patients from the same physician in the two treatments arms will adjust for "physician effect." Contamination will also be low because there is relatively minimal involvement required of PCPs in CAMEO. In addition, randomization will be stratified by depression status (yes or no) and prior substance abuse treatment (yes or no) to avoid potential imbalance of these factors between the two arms. Xinli Li, PhD, MS (statistician) will provide a randomization chart to randomize patients in blocks of 3 and 6.

D9a. <u>Sample size</u>

Our sample size is calculated based on estimated intervention effects on the primary outcome; the Brief Pain Inventory (BPI) total score. The BPI total score is continuous measure: a 0-10 scale and reflects the average of the BPI pain severity and interference scores. The SCAMP trial[40;98] showed that the standard deviation was 2.44 for the BPI total score. We will assume this is the common standard deviation across the two treatment arms. A between-group treatment difference of 1 point in the BPI total score represents a minimum clinically meaningful intervention effect (i.e. 0.3 SD effect size).[99] With a two-sided test at alpha=0.05, we will have 80% power to detect a 1-point difference in BPI score with 135 subjects in each arm. With a conservative 12% attrition, we will then need (135*2)/0.88 = 300 subjects (150 in each arm). Because of clinical equipoise, it is not clear which treatment approach will produce the most meaningful differences in outcomes. Therefore, the

null hypothesis of no clinically significant difference between the two active treatment arms would be satisfied given a treatment effect size < 0.3 standard deviation. We expect both active treatment arms to significantly improve pain intensity, function, and other relevant outcomes at 6 and 12-months compared to baseline.

D9b. Data analysis: Baseline Comparability

Because of the size of this study, it is expected that randomization will produce treatment groups that are comparable and balanced. To test this assumption, we will tabulate baseline characteristics of the two trial arms for potential imbalance in variables such as socio-demographic variables, medical and psychiatric comorbidity, duration of back pain, and current and prior pain treatments. Continuous variables will be assessed with graphical displays and summary statistics (means, standard deviation, range, etc). Frequency distributions and percentages will be calculated for categorical data.

D9c. Primary outcomes at each time point and mixed-effects models for repeated measures

We will summarize this primary outcome at each time point (3, 6, 9 and 12 months) for both study arms. The difference between time points will be compared between the two treatment arms. To compare the primary outcome at each time point relative to baseline within each arm, paired T-test or Wilcoxon rank-sum test will be used. Between-arm comparisons will be based on similar statistical tests. The primary endpoint will be at 6 months. Secondarily, "early" response will be assessed at 3 months and "sustained" response at 9- and 12-months. We plan an intent-to-treat analysis approach.

Since the primary outcome is measured repeatedly at baseline, 3, 6, 9, and 12 months, we will also fit linear (for continuous variables) or non-linear (for categorical variables) mixed-effects models to data at all time points, which accounts for the correlation of measurements from the same subject/physician and allows adjustment of confounding factors. To illustrate the idea, BPI total score is denoted by Y_{ik} of the kth measure of subject i. We will fit the following model:

$$\log it(Y_{ik}=1) = X_{ik}\beta + \alpha_{i0} + \alpha_{i1}t_{ik} + \lambda_j,$$
$$\text{where } \alpha_{i0} \sim N\left(0,\ \varepsilon^2\right), \alpha_{i1} \sim N\left(0,\ \delta^2\right), \lambda_j \sim N\left(0,\ \phi^2\right).$$

Here the vector X_{ik} represents fixed covariates for patient i at time k, and β represents the corresponding coefficients. The fixed covariates will include critical covariates such as treatment arm, demographics, and other patient characteristics. α_{i0}, α_{i1} and λ_j represent the random effects, which are the patient-specific intercept, patient-specific slope and the physician-specific intercept (j index the physician). The fit of the model will be assessed using graphical techniques and regression diagnostics. We will also examine potential interaction terms among the covariates, particularly interactions between each treatment arm and other covariates. If there is a significant treatment effect, we will report the 95% confidence interval of the treatment difference after adjusting for a pre-specified set of baseline covariates. The model will be fitted by SAS procedures MIXED and NLMIXED. All statistical Analysis Software (SAS, version 9.1, Cary, N.C.).

D9d. Missing data

Missing data are unavoidable in longitudinal studies. Based on our prior studies, we anticipate a possible dropout rate between 8% and 12%, which is reflected in the sample size calculation. We will compare patient demographic characteristics between those who stay in the study and those who drop out to examine whether or not there are characteristics that discriminate drop-outs and non-drop-outs. It is possible that the dropout mechanism does not depend on unobserved outcomes (Missing At Random, or MAR),[100] where no bias will be introduced by ignoring the missing-data mechanism. We can simply use all observed outcomes for the analysis. Under circumstances where power loss is of concern, we will use multiple imputation procedures to make use of all relevant observed variables to enhance the power. The SAS procedure MI and MIANALYZE will be used for multiple imputation.In case drop-outs are Missing Not At Random (MNAR), which means the likelihood of drop-out depends on un-observed outcome, potential bias can be introduced if the miss-data mechanism is ignored. We will make various assumptions regarding the missing-data process based on the best of our knowledge. With these assumptions, we will fit proper models, either in the form of selection model,[101] pattern mixture model,[102] or latent variable model to account for the missing-data process. A sensitivity analysis will be conducted to compare the results based on different assumptions and models and to assess the robustness of the inference.

D9e. Secondary Analyses

We will employ several secondary analyses to assess the effectiveness of the PHARM and BEH treatment arms compared to each other in improving:

- Patient global impression of change
- Health-related quality of life
- Opioid dose

Analytic techniques will be similar to those previously described. Since there will be a number of secondary outcomes, it presents a multiple-comparison situation. We will use Bonferroni approach to adjust for statistical significance threshold when the number of tests is less than 20. Otherwise, we will use the false discovery rate (FDR)[103] to control the magnitude of false positives.

We will also stratify our analysis based on a baseline depression status (PHQ-15) and prior treatment for substance abuse disorder (yes or no on the eligibility screener). Our previous research indicated that 30% to 40% of patients will have clinically significant depression, which adversely affects pain outcomes. Thus, depression is a potential moderator and prevalent enough to warrant stratification. In addition, since substance abuse (including opioids) is relatively common in chronic pain populations and poses unique management challenges, we will further stratify our analysis by prior treatment for a substance abuse disorder.

D9f. Economic evaluation

We will conduct a cost-consequences analysis to determine changes in health care utilization that may offset the intervention costs between treatment arms. To conduct this analysis, we will use established methods [104] to estimate direct costs of the interventions and health care spending for study participants during the 12-month enrollment period from the perspective of the VA. Sensitivity testing, according to guidelines from VA Health Economics Research Center (HERC), will be performed to account for assumptions, including changes in intervention costs and changes in costs related to inpatient or outpatients services.

D9g. Costing

We will use both micro- and gross (average) costing method [105;106] to estimate intervention and health care utilization costs, respectively. Applying HERC guidelines, we will measure intervention-related activities and their associated costs.[104] These intervention costs will include: 1) medication costs for patients; 2) estimates of overall pharmacy costs from the DSS pharmacy file for both study arms; 3) study nurse and psychologist costs derived from time and activity logs that the they will complete for each intervention-related activity (preparation for and delivery of interventions; attempted and completed calls to patients; patient assessment, education, and counseling; record keeping and review after intervention contacts; and communication between them and PCPs); 4) Nurse and psychologist average salaries plus fringe benefits; 5) supervising intervention physicians/ pharmacist time; 6) study materials provided to intervention patients; and 7) overhead.

We will use VA DSS data to obtain health care utilization and cost estimates of trial patients. From the VA Patient Treatment File (PTF), we will collect hospital discharge date, days of hospital stay in each treating bed section/specialty and ICD-9 diagnoses (especially primary or secondary diagnoses) of low back pain assigned to each stay and determine the cost per admission. From the Outpatient Care File (OPC), we will collect dates of any outpatient visits for low back pain, location of care (stop code), CPT codes assigned to each visit, and the type of provider delivering care. We will classify all visits into primary care, emergency, pain, specialty medicine or surgery, mental health or substance use disorder, and other treatments based on clinic identifiers.

The number of outpatient prescriptions, especially for analgesic and psychotropic medications, will be found in the DSS NDE Pharmacy Datasets from VA Information Resource Center (VIReC)[107] to determine the cost per prescription. Laboratory and radiographic testing will be collected to determine the number of tests for low back pain evaluation and the costs per test. The cost of other care will be obtained from utilization data included in VA Austin databases. For non-VA care, covered by the VA, fee basis files will be merged into DSS data[108] For non-VA care not covered by the VA, we will we rely on patient self-report of outpatient visits and hospitalizations. Since VA utilization data does not include cost estimates, the above mentioned data sets will be merged to the HERC average cost datasets to estimate the costs associated with VA utilization for each participant.[109;110] Costs will be converted to a standardized year using the Consumer Price Index (CPI). The CPI is calculated on the basis of 305 items representing all goods and services purchased for everyday living

by all urban residents. We will use the general CPI for primary analysis and the Gross Domestic Product Implicit Price Deflator for a sensitivity analysis.

D9h. Analysis

The aim of the cost consequences analysis will be to compare intervention and health care costs for each study arm. The dependent variable will consist of primary cost outcomes calculated from the VA perspective. Having obtained the relevant VA healthcare utilization events, the healthcare event requires **valuation**, which is the task of assigning a reasonable market-level dollar for the expense amount. For each patient, outpatient events (visits, procedures, labs, medications, etc.) and inpatient DRGs and medical events (procedures, labs, medications, etc.) are captured from these patient-specific administrative datasets. VHA databases provide sufficient outpatient and inpatient procedure and associated treatment classifications (by CPT-4, DRG and ICD-9 codes) to allow valuation and this has been accomplished at the VA's Health Economics Resources Center. Dr. French has created similar datasets in other VA funded studies and publications. For health care utilization and the associated costs, we will compare inpatient days, outpatient visits, telephone care, number of prescriptions and radiographic. However, we may not be able to include inpatient costs because hospitalization may occur rarely during the trial which, coupled with high inpatient costs, makes estimates of between-group differences imprecise.

The two study arms will be specified as two sets of independent binary control variables (Arm 1, Arm 2); Arm 1: =1 for pharmacological (PHARM), =0 otherwise; Arm 2: =1 for behavioral (BEH), =0 otherwise; The model will be adjusted for comorbidities, age, and other potential confounders. Univariate, bivariate and regression techniques for repeated measures will be used to estimate the healthcare events and expenditure amounts. Regression diagnostic techniques will be used to determine if the assumptions of independence, normality and homoskedasticity are met for the cost values. Log transformation of healthcare expenditure dependent variables is common due to skewed distributions. Multicollinearity will be assessed through an examination of the zero-order correlation matrix and by examination of condition number bounds. Residuals will be analyzed to identify outliers. In some analyses, the dependent variables of VA expenditures per episode of care will be used in regressions and may require a variable transformation to normalize the dependent variable's distribution. In this case we will use an econometric transformation, the inverse hyperbolic sine (IHS) transformation: $Z = \log [2Y + (2^2Y^2 + 1)^{½}]/ 2$. Note: This transformation is defined for all real numbers including zero, negative values, and positive values, making it appropriate for use with dollar values.

Costs will be reported in current year's dollars. Sensitivity analyses will be used to account for assumptions, including changes in intervention costs and changes in costs related to inpatient or outpatients services. If the interventions are found to be differentially more effective and costly, we will perform a cost-outcome analysis (i.e., incremental cost-effectiveness). We will estimate the effect of the interventions on the primary outcome (BPI total score). The incremental cost to achieve a clinically meaningful decrease in BPI total scores due to the interventions, i.e., the cost-effectiveness ratio, is calculated as the difference in intervention costs between the two treatment arms, divided by the difference in effectiveness between groups.

$$\frac{\text{Intervention Costs} + (\Delta \text{ Health Care Costs})}{(\Delta \text{ BPI}_{\text{PHARM Treatment}})-(\Delta \text{ BPI}_{\text{BEH Treatment}})}$$

where Δ BPI = change in BPI score, and Δ Health care costs = difference two groups

D10. PROJECT MANAGEMENT PLAN

D10a. Project Timeline

The 1st quarter in year 1 will involve hiring and training personnel. Important steps will include: (1) finalizing treatment algorithms; (2) training the study nurses in assessing pain, and opioid and analgesic management; (3) training clinical psychologists in delivering pain self-management/coping skills; (4) training the research assistant in screening, enrolling, and consenting study participants; (5) programming the electronic medical records to identify potential study subjects based on low back pain diagnosis and opioid treatment; and (6) obtaining permission from individual treating physicians to approach patients of theirs who might be eligible. During the next 2.5 years, we will enroll 300 participants (randomizing 135 each group: 1) pharmacological; and 2) behavioral treatment. Enrollment will average 4 new participants per week (10 per month). The 300 participants will be treated for 6 months. Participants will have outcome assessment at baseline, 3, 6, 9, and 12 months. Thus, enrollment will be conducted from the 2nd quarter (year 1) until the 4th quarter (year 3); the

intervention phase from 2nd quarter (year 1) until 3rd quarter (year 4); and outcome assessments from 2nd quarter (year 1) until the end of study period. Baseline analysis will begin at the beginning of year 4 and conducted during the final 12 months of the study (separate baseline, 6 month, and end-of-study analyses). Main reports and manuscripts will be prepared in the 3rd and 4th quarters of Year 4. As shown in the gannt chart below, this is a 4-year project.

CAMEO Study Timeline

	Year 1				Year 2				Year 3				Year 4			
Quarters	1	2	3	4	1	2	3	4	1	2	3	4	1	2	3	4
Start-up	■															
Enrollment		■	■	■	■	■	■	■	■	■	■	■				
Intervention		■	■	■	■	■	■	■	■	■	■	■	■	■		
Assessments		■	■	■	■	■	■	■	■	■	■	■	■	■	■	■
Analysis													■	■	■	■
Manuscripts															■	■

D10b. Overall project coordination and facilities

Overall project coordination will be led by Dr. Bair (PI) and Ms. Sargent (Project coordinator). Ms. Sargent will coordinate all aspects of the project, trouble-shoot any problems, and provide regular updates on recruitment progress. We have secured commitments from the appropriate clinical services to conduct the study (see included letters of support). Drs. Bair, Krebs, Damush, Zillich, French, and Kroenke have office, meeting and research space within the HSR&D Center for Implementing Evidence-based Practice (CIEBP) at the RVAMC. Dr. Shen's office is within the Health Information Technology Services building, a 5-minute drive from CIEBP.

D10c. Investigator roles

The proposed project will be conducted by a strong multidisciplinary team with extensive experience in areas relevant to this study. Specific roles of the research team are outlined in more detail in the budget justification. The efforts of each of the investigators will be coordinated by Dr. Bair and Ms. Sargent to develop plans, review progress, discuss analysis results, and set priorities for the research team. Matthew Bair, MD, MS, has expertise in pain interventions in primary care and chronic pain and depression comorbidity. He will serve as PI and provide overall study direction. Erin Krebs, MD, MPH, is a health services-investigator with expertise in the safe and effective use of opioids. She will serve as Medical Director for the study and co-lead (with Dr. Bair) the weekly care management meetings. Kurt Kroenke, MD, is internationally known for his expertise in symptoms and mental health research in primary care and will contribute senior guidance on all phases of the study. Teresa Damush, PhD, will oversee the delivery of the pain self-management/coping skills intervention to patients and will train the psychologists to administer the program. Alan Zillich, PharmD, is a recipient of a HSR&D CDA-2 entitled "Implementation Strategies to Improve Prescribing of Pharmaceuticals." He will contribute his expertise in the safe and effective use of analgesics for intervention patients. Dustin French, PhD, is a health economist-investigator with extensive experience in medication safety and VA large database research. Dr. French will guide the economic analysis. Zhangsheng Yu, PhD, is an experienced clinical trial biostatistician who will lead the data analysis. Additional analytic support will be provided by James Slaven, MS, who specializes in hierarchical and longitudinal analyses. The CAMEO nurse care managers will be Carol Kempf, RN and Sharon Weitlauf, RN, both experienced nurse care managers in our previous trials. Jennifer Lydon-Lam, PhD, Samantha Outcalt, PhD, and Shannon Woller, PsyD will deliver the behavioral intervention. Christy Sargent, BA, will serve as project coordinator, Amanda Gerwig, BS as the research assistant, and Tenesha Pennington, MS as the data manager. Our study consultant is Rollin "Mac" Gallagher, MD, MPH who is the VA's Deputy National Program Director for Pain Management, immediate-past President of the American Academy of Pain Medicine, and an internationally-recognized pain specialist with particular expertise in algorithm-based therapy with co-analgesics, opioid therapy, and integrating pain medicine and primary care.

D10d. Data Management

Each of the study questionnaires will be programmed into a desktop computer using Microsoft Access. The research assistant will interview the patient and enter data simultaneously into the Access program. Within the Access program, algorithms will be created to check for inappropriate or missed data entry. Computer algorithms will also automatically score the questionnaires and store the summary scales within the same database, as well as determine the appropriate date for follow-up interviews. These data will be backed up daily onto a secured server at RVAMC. Participant social security numbers, names, addresses and other personal information will be restricted to authorized personnel to protect confidentiality. For data analysis and other uses of the data, this information will be removed from the database and be replaced with a simulated identification number. This strategy has been used in our previous trials to screen and enroll patients, accurately complete follow-up assessment, and protect patient privacy. We have experience in setting up data integrity protocols and data back-up to minimize the risk for lost or inaccurate data.

D10e. Data Safety Monitoring Plan

The following multi-component Data Safety Monitoring Plan has been used in our VA RR&D-funded trial of OEF/OIF veterans with chronic musculoskeletal pain and NIMH-funded pain/depression SCAMP trial, all of which have enrolled veterans. Subjects are monitored during study interventions in the following ways: 1) Frequent Subject Contacts by Nurse Care Manager (NCM) and Clinical Psychologists. All subjects are closely followed by scheduled NCM telephone contacts. Response to medications as well as side effects from them are closely monitored. All interview responses are part of the study database. 2) Weekly Case Management Review. The NCMs meet weekly with the supervising physicians and a doctor of pharmacy to review the previous week's subject contacts. Medication management as well as any side effects or problems are reviewed and discussed. Minutes from these meetings are prepared and stored electronically on a desktop hard drive with server back-up. The study PI and physician co-investigators are available by pager at all times. 3) Monthly Executive Committee Meetings. The PI and co-investigators meet monthly for Executive Committee meetings. At these meetings the investigators discuss recruitment, subject safety, protocol adherence, and any other issues that may arise. Minutes are kept and are circulated to any co-investigators not able to attend. Subjects will be allowed to withdraw from the trial at any time. If a patient withdraws, we will determine the reason for withdrawal. Possible reasons will include: 1) death, 2) worsening of comorbid medical conditions making follow-up impossible, 3) medication side effects, and 4) other. If subjects withdraw, we will attempt to obtain their permission to complete the remaining outcome assessments. Data will be analyzed on an intent-to-treat basis. We will establish a local Data Safety Monitoring Board (DSMB) that meets every 6 months during the study and is notified via email of all deaths and SAEs at the time of local IRB notification. Members will consist of non-study investigators and will include a pain specialist, a general internist, a clinical trialist, a nurse, and a biostatistician.

The DSMB will monitor the following: (a) subject recruitment, accrual, and retention; (b) patient outcomes and adverse events; (c) subject safety, privacy, and confidentiality procedures; (d) diversity of subject enrollment (i.e., gender, race and ethnicity) in terms of concordance with NIH targeted enrollment table for this trial; (e) data quality and major findings in terms of treatment benefits and risks; (f) results of related studies that impact subject safety; (g) assessment of scientific reports that might alter the benefit/risk ratio of the study. Analyses of data will be performed by the study biostatistician but will be independently interpreted by the DSMB (which will include an independent biostatistician) which can request additional analyses as the DSMB members see fit. Since this is an effectiveness trial using evidence-based treatments, there are no pre-planned interim analyses or stopping rules. The DSMB will submit the report of its twice-a-year meetings to the study investigators who will in turn report the information to the IRB in its continuing review, unless there are items the DSMB feels should be reported to the IRB immediately.

D11. DISSEMINATION AND IMPLEMENTATION PLAN

The VHA National Pain Management Strategy Coordinating Committee will serve as our primary channel for disseminating study findings to VA providers, administrators, researchers and policy makers. Findings will be disseminated to the committee in the form of summary reports and presentations given either in the monthly conference calls or at their annual face-to-face meeting (or both). The Committee will advise and coordinate next steps for dissemination, including dissemination to other relevant entities such as VISN and hospital administrators; local Pain Management Committees; the VISN Pain Points of Contact, the Office of Quality and

Performance; and the national working groups related to pain management education, guideline development, and performance measures. Additionally, a summary of study findings and implications will be posted on the VHA Pain Management Committee's website in a format readable to veterans. Findings will be disseminated to our research audiences through scientific presentations and publications, and HSR&D cyber seminars, as well as through conference calls with the Pain Research Working Group, a subcommittee of the VHA National Pain Management Strategy Coordinating Committee. We also will seek synergistic opportunities with the Pain Research, Informatics, Medical comorbidities and Education (PRIME) Center directed by Dr. Robert Kerns (VA National Program Director for Pain Management).

Other resources include a VA national pain management website (www.va.gov/pain_management); a widely subscribed VA pain list serve; monthly national educational teleconferences targeting providers and administrators; a network of VISN Pain Points of Contact who hold monthly teleconferences and who serve an important liaison role between the National Pain Management Committee and facility level pain committees; and a network of VA and non-VA pain-relevant investigators (the Pain Research Working Group) who hold twice monthly teleconferences and yearly face-to-face meetings and who, among their goals, work to promote dissemination of research findings and to influence practice and policy related to pain care. In sum, an established network of resources is already in place to disseminate study findings. In addition, to the local, regional, and national pain groups we are already tied into, we will disseminate study findings to the Mental Health and Substance Use Disorder QUERI groups, the National Serious Mental Illness Treatment Research and Evaluation Center (SMITREC), the Mental Illness Research, Education, and Clinical Center (MIRECC), and the HSR&D Center for Information Dissemination and Education Resources (CIDER). Our research group has disseminated study findings in the VA HSR&D Cyber-seminar forum sponsored by CIDER.

D12. POTENTIAL LIMITATIONS OF PROPOSED STUDY

Study design: Our research team thoroughly discussed and debated study design issues. We agreed with the reviewers that substantial study design changes were necessary to frame as a comparative effectiveness trial which was adequately powered. Our previous proposal outlined a 3-arm trial (pharmacological vs. behavioral vs. attention control). However, findings from recent pain trials (SCAMP and APT) demonstrated the ineffectiveness of usual care, making an attention control of less relevance. A 3-arm trial comparing combined treatment (PHARM + BEH) vs. PHARM vs. BEH might be warranted pending the results of our head-to-head comparison of each single-mode intervention. Such a 3-arm study would require more than one VAMC to enroll an adequate number of subjects and would be best informed by the results of our clinical and cost-effectiveness findings from the present 2-arm trial.

Generalizability: The study sample will be drawn from a single, VA medical center and two VA community based outpatient clinics in Indiana. As such, the sample may not be representative of all patients with CLBP on long-term opioids. However, our treatment model could certainly be applied in other VAMCs/CBOCs.

Innovation: A core aspect of the CAMEO interventions is nurse care management which has been found to be effective in other chronic illnesses. While it could be argued that nurse care management is not all that novel, its use for chronic pain generally and opioid management specifically is innovative and holds promise for improving opioid management in primary care. Furthermore, testing the comparative effectiveness of different treatments (pharmacological vs. behavioral) administered by different types of clinicians embedded in VA primary care (nurses vs. psychologists) is both novel and important.

D13. STRENGTHS AND SYNOPSIS OF STUDY

Despite study limitations, the CAMEO trial has a number of strengths, including: (1) *testing the comparative effectiveness of two unique interventions designed to improve the management of CLBP*; (2) management approaches that challenge existing treatment paradigms for chronic pain care and have the potential to be applied across multiple VA clinical settings; (3) a high interest study population that has been frustrating to providers; (4) a randomized clinical trial design; (5) ample statistical power to detect meaningful differences in our primary outcomes; and (6) an economic evaluation that may provide VA administrators, clinical managers, and policy makers with data to inform budget decisions to invest in these interventions and make them routinely available to veterans suffering from chronic low back pain.

Should the interventions prove effective in reducing pain intensity and interference with activities our next step will be: (1) to conduct a post-study, summative evaluation using qualitative methods. The qualitative study will consist of semi-structured interviews with patients purposefully sampled from each arm of the trial to evaluate

which aspects of CAMEO are most effective as perceived by patients. Instead of proposing a nested qualitative study as part of this application, we will apply for either a separate HSR&D pilot grant or internal grant funding. This additional funding would allow us to conduct a more rigorous, in-depth qualitative study to complement CAMEO. We have successfully obtained supplemental funding on two previous occasions and have found this type of work to be highly informative in elucidating trial results.

In sum, PCPs are faced with numerous challenges in treating patients with chronic back pain, including managing the complexities of opioid therapy while trying to ensure against abuse and addiction. The interventions being tested in the CAMEO trial have the potential to provide primary care settings with new treatment models that will help to guide PCPs while at the same time providing much needed relief for veterans suffering from chronic back pain.
