# Supplementary material for: Effect of Medication Optimization vs Cognitive Behavioral Therapy Among US Veterans With Chronic Low Back Pain Receiving Long-term Opioid Therapy: A Randomized Clinical Trial
Source: JAMA Netw Open. 2022 Nov 17;5(11):e2242533. doi: 10.1001/jamanetworkopen.2022.42533 (PMC9672973; doi:10.1001/jamanetworkopen.2022.42533)
Supplement: Supplement 2. — eTable 1. List of Analgesics Assessed on Medical Record Review eTable 2. Prescribed Opioid Morphine Milligram Equivalent (MME) Data eTable 3. Number of Prescribed Analgesics Comparison [file jamanetwopen-e2242533-s002.pdf]

Supplementary Online Content

Bushey MA, Slaven JE, Outcalt SD, et al. Effect of medication optimization vs cognitive behavioral therapy among US veterans with chronic low back pain receiving long-term opioid therapy: a randomized clinical trial. *JAMA Netw Open*. 2022;5(11):e2242533. doi:10.1001/jamanetworkopen.2022.42533

eTable 1. List of Analgesics Assessed on Medical Record Review
eTable 2. Prescribed Opioid Morphine Milligram Equivalent (MME) Data
eTable 3. Number of Prescribed Analgesics Comparison

This supplementary material has been provided by the authors to give readers additional information about their work.

© 2022 Bushey MA et al. *JAMA Network Open*.

eTable 1. List of Analgesics Assessed on Medical Record Review

Non-Steroidal Anti-Inflammatory Drugs (NSAIDs)	Ibuprofen, Naproxen, Etodolac, Diclofenac, Meloxicam, Salsalate, Sulindac, Aspirin (≥325mg), Celecoxib
Muscle Relaxants	Cyclobenzaprine, Methocarbamol, Tizanidine
Antidepressants	Amitriptyline, Nortriptyline, Duloxetine
Gabapentinoids	Gabapentin, Pregabalin
Opioids	Hydrocodone, Oxycodone, Morphine, Methadone, Fentanyl, Tramadol, Codeine
Topical	Capsaicin, Lidocaine, Menthol-salicylate, Diclofenac gel
Supplements	Glucosamine, Hyaluronic acid (oral)
Other	Acetaminophen

eTable 2. Prescribed Opioid Morphine Milligram Equivalent (MME) Data

	**MED Group**					**CBT Group**				
Timepoint	**BL**	**3mo**	**6mo**	**9mo**	**12mo**	**BL**	**3mo**	**6mo**	**9mo**	**12mo**
Mean	68.6	64.5	61.2	59.4	54.1	76.2	70.8	72.7	70.9	66.7
SD	81.0	73.7	75.2	77.1	73.3	96.5	94.7	96.8	97.6	96.1
Median	40	40	40	30	30	45	40	40	40	40
Mode	40	40	40	30	0	30	30	30	30	30
Max	450	450	450	480	450	900	900	900	900	900
Min	8	0	0	0	0	10	0	0	0	0
Q1	23	20	20	20	15	30	30	30	30	20
Q3	69	71	71	69	60	90	90	90	90	90
Skew	2.7	2.6	2.8	2.9	2.9	5.4	5.7	5.4	5.3	5.5
Kurtosis	7.5	8.0	8.8	9.8	9.7	41.2	45.8	41.5	40.4	43.8
[a] BL compare	n/a	.67	.20	.07	0.006	n/a	.38	.51	.25	0.06
[b] Group compare 1	0.11	.30	.04	.06	0.06	0.11	.30	.04	.06	0.06
[c] Group compare 2	0.48	0.55	0.28	0.29	0.24	0.48	0.55	0.28	0.29	0.24

[a] BL compare = P-value of within group comparison to baseline values by Mann-Whitney U test.
[b] Group compare 1 = P-value of between group comparison at each timepoint by Mann-Whitney U test.
[c] Group compare 2 = P-value of between group comparison at each timepoint by Student's T-Test.

© 2022 Bushey MA et al. *JAMA Network Open*.

**eTable 3.** Number of Prescribed Analgesics Comparison										
	**MED Group**					**CBT Group**				
Timepoint	**BL**	**3mo**	**6mo**	**9mo**	**12mo**	**BL**	**3mo**	**6mo**	**9mo**	**12mo**
Mean	0.3	2.2	1.7	1.4	1.3	0.3	0.8	0.7	0.7	0.8
SD	0.6	1.3	1.3	1.1	1.1	0.6	1.0	0.8	0.9	1.0
Median	0	2	2	1	1	0	0	1	0	1
Mode	0	3	2	1	1	0	0	0	0	0
Max	3	6	6	6	4	3	4	3	3	5
Min	0	0	0	0	0	0	0	0	0	0
Q1	0	1	1	1	0	0	0	0	0	0
Q3	1	3	3	2	2	1	1	1	1	1
Skew	1.8	0.3	0.5	0.7	0.5	2.0	1.2	1.0	1.1	1.5
Kurtosis	3.0	-0.2	0.0	0.8	-0.4	3.7	0.9	0.4	0.5	2.8
[a] BL compare	n/a	< .001	< .001	< .001	< .001	n/a	< .001	< .001	< .001	< .001
[b] Group compare 1	0.76	< .001	< .001	< .001	< .001	0.76	< .001	< .001	< .001	< .001
[c] Group compare 2	0.71	< .001	< .001	< .001	< .001	0.71	< .001	< .001	< .001	< .001

[a] BL compare = P-value of within group comparison to baseline values by Mann-Whitney U test.
[b] Group compare = P-value of between group comparison at each timepoint by Mann-Whitney U test.
[c] Group compare 2 = P-value of between group comparison at each timepoint by Student's T-Test.

© 2022 Bushey MA et al. *JAMA Network Open*.
