# Supplementary material for: Effect of Medication Optimization vs Cognitive Behavioral Therapy Among US Veterans With Chronic Low Back Pain Receiving Long-term Opioid Therapy: A Randomized Clinical Trial
Source: JAMA Netw Open. 2022 Nov 17;5(11):e2242533. doi: 10.1001/jamanetworkopen.2022.42533 (PMC9672973; doi:10.1001/jamanetworkopen.2022.42533)
Supplement: Supplement 3. — Data Sharing Statement [file jamanetwopen-e2242533-s003.pdf]

Data Sharing Statement

Bushey. Effect of Medication Optimization vs Cognitive Behavioral Therapy Among US Veterans With Chronic Low Back Pain Receiving Long-term Opioid Therapy. *JAMA Netw Open*. Published November 17, 2022. doi:10.1001/jamanetworkopen.2022.42533

Data
Data available: No
